# Association between dietary niacin intake and diabetic retinopathy in a Catalonian population: a cross-sectional study

**DOI:** 10.3389/fnut.2025.1626379

**Published:** 2025-10-14

**Authors:** Jeimy Katherine Torres-Méndez, Marina Idalia Rojo-López, Maria Antentas, Pau Vendrell, Emilio Ortega, Nuria Alonso, Esmeralda Castelblanco, Andrea Muscarà, Esther Rubinat, Nuria Alcubierre, Marta Hernández, Joana Rossell, Minerva Granado-Casas, Didac Mauricio, Josep Julve

**Affiliations:** ^1^Group of Endocrinology, Diabetes & Nutrition, Institut de Recerca Sant Pau (IR Sant Pau), Barcelona, Spain; ^2^Department of Medicine, Universitat Autònoma de Barcelona, Barcelona, Spain; ^3^Grup de Diabetis d‘Atenciò Primària (DAP-Cat), Unitat de Suport a la Recerca Barcelona, Fundaciò Institut Universitari per a la Recerca a l'Atenció Primària de Salut Jordi Gol i Gurina, Barcelona, Spain; ^4^Department of Medicine, Universitat de Barcelona, Barcelona, Spain; ^5^Department of Endocrinology and Nutrition, Institut d'Investigacions Biomèdiques August Pi i Suñer, Hospital Clínic, Barcelona, Spain; ^6^Center for Biomedical Research in the Physiopathology of Obesity and Nutrition (CIBEROBN), Instituto de Salud Carlos III, Madrid, Spain; ^7^Center for Biomedical Research on Diabetes and Associated Metabolic Diseases (CIBERDEM), Instituto de Salud Carlos III, Madrid, Spain; ^8^Department of Endocrinology and Nutrition, University Hospital Germans Trias i Pujol, Badalona, Spain; ^9^Department of Internal Medicine, Endocrinology, Metabolism and Lipid Research Division, Washington University School of Medicine, St. Louis, MO, United States; ^10^Department of Clinical and Experimental Medicine, University of Messina, Messina, Italy; ^11^Department of Nursing and Physiotherapy, University of Lleida, Lleida, Spain; ^12^Lleida Institute for Biomedical Research Dr. Pifarré Foundation, IRBLleida, University of Lleida, Lleida, Spain; ^13^Avantmedic Center, Lleida, Spain; ^14^Department of Endocrinology & Nutrition, University Hospital Arnau de Vilanova, Lleida, Spain; ^15^Department of Endocrinology & Nutrition, Hospital de la Santa Creu i Sant Pau, Barcelona, Spain; ^16^Department of Medicine, University of Vic – Central University of Catalonia, Vic, Spain

**Keywords:** retinopathy, diabetes, vitamin B3, niacin, neuropathy, microangiopathy, tryptophan

## Abstract

**Background:**

Accumulating evidence suggests that niacin interventions may have a beneficial role in preventing ophthalmological microangiopathic remodeling. This study aimed to assess the impact of niacin intake on the presence of diabetic retinopathy (DR) in two independent Mediterranean cohorts of individuals with type 1 diabetes (T1D) and type 2 diabetes (T2D).

**Methods:**

Cross-sectional, population-based epidemiologic study. A total of 243 individuals with T1D, and 291 individuals with T2D. All participants underwent an eye examination. Dietary niacin intake was analyzed using a validated Spanish 101-items food frequency questionnaire consumption. The association between niacin intake and DR was assessed using a multivariate logistic regression, with potential non-linear associations further explored through restricted cubic spline regression. DR diagnostic was established via multifield stereoscopic retinal photography, in accordance with the international consensus on DR.

**Results:**

DR was identified in 103 out of 243 individuals with T1D and 144 out of 291 with T2D. Dietary niacin intake did not differ within the study groups according to DR status. Multivariate logistic regression models also revealed no association between dietary niacin intake and DR. As previously described, the duration of diabetes for T1D and T2D, [OR (95%CI), 1.02 (1.01–1.03), and 1.02 (1.02–1.03), respectively] and glycated hemoglobin (HbA1c) [OR (95%CI), 1.16 (1.09–1.24), and 1.14 (1.09–1.18), respectively] were identified as the main determinant variables for DR in both groups.

**Conclusions:**

We found that dietary niacin intake was not associated with the presence of RD in subjects with either T1D or T2D. Further research is needed to better understand the potential role of niacin in the development or prevention of DR.

## 1 Introduction

Diabetic retinopathy (DR) is a chronic and progressive eye condition affecting approximately one-third of subjects with diabetes mellitus (DM) (1). Epidemiologically, DR is strongly linked to prolonged duration of DM, hyperglycemia and hypertension (2). The prevalence of DR is higher in about 75% of subjects with type 1 diabetes mellitus (T1D) compared with type 2 diabetes mellitus (T2D), which is approximately 25% of total (3). If untreated, DR can progress into several disease stages including different degrees of proliferative DR, caused by an abnormal growth of retinal microvasculature, as well as diabetic macular edema (4–7). DR is currently a leading cause of irreversible vision impairment and blindness, occurring in about 25% to 30% of affected subjects (1, 2).

Clinically, microangiopathic complications of DM include, among others, DR, which is considered a neurovascular complication. DR can be detected and classified after an accurate eye examination (8); however, DR has been postulated to progress in a subclinical manner (9). The pathogenesis of DR is complex and likely multifactorial, involving altered intricate metabolic and cellular processes that ultimately result in damage to the retinal vasculature and retinal nerve tissue (10). Actually, recent studies indicate that subclinical neuroinflammation, triggered by hyperglycemia, may precede vascular remodeling (6).

In both T1D and T2D, dietary management and physical activity play a pivotal role in the prevention of DR. In T1D, these lifestyle measures are additionally combined with carbohydrate counting and intensive insulin therapy to optimize glycemic control (11). However, and beyond glycemic control optimization, no specific strategies exist to efficiently protect against early subclinical stages of incident DR (12). Therefore, current research has been focused in uncovering novel differentially expressed molecular biomarkers to sense subclinical phases of this disabling complication.

The reduction of the oxidized form of nicotinamide adenine dinucleotide (NAD+) in nerve tissue has been related to the induction of experimental neuroinflammation (13–15) and neurodegeneration (16, 17). Furthermore, accumulating data supports the notion that supplementation with NAD+ precursors, increases intracellular NAD+ availability and protect against neurodegeneration in experimental mice of retinal eye disease (18–24), retinal vascular remodeling (25), and in the treatment of photoreceptor degeneration following DR (26) as well as other disorders with a neurodegenerative component (27, 28).

In observational studies, dietary niacin derivatives, which include tryptophan, an essential amino acid for *de novo* synthesis of niacin (29), are commonly estimated using a validated food frequency questionnaire consumption (FFQC). Accordingly, dietary niacin equivalents (NEq) are considered as a more accurate measure of estimated daily niacin consumption (30, 31), to analyze associations with DR in human cohorts. Importantly, such nutritional questionnaires are not frequently evaluated in most study cohorts on DR. Therefore, there are few clinical studies directly assessing the potential relationship between niacin intake and DR development. On the one hand, it has been found that the circulating concentrations of tryptophan are reduced in subjects with DR (32). Furthermore, niacin has been reported to improve vasodilatation and visual acuity in treated subjects with diagnosed chronic retinal vein occlusion (33), a condition that is frequently increased in subjects with DM and may be diagnosed along with DR (34). In addition, cystoid macular edema (CME) is a consequence of DR; however, some cases have also been reported in association with high-dose niacin treatment (35).

In this context, we hypothesized that an increased intake of niacin, or NEq, may have the potential to delay or prevent incident DR. Therefore, we evaluated the association between niacin intake levels, NEq intake, and DR in two independent cohorts of individuals with T1D and T2D.

## 2 Methods

### 2.1. Study design and setting

The current cross-sectional substudy was undertaken in 2 independent cohorts of subjects with T1D and T2D from Catalonia, northeast Spain, recruited between 2010 and 2014. Comprehensive details on the 2 cohorts are available in earlier publications (36–38).

### 2.2. Study subjects

In the substudy, a sample of 534 participants was available: 243 participants with T1D and 291 with T2D (Supplementary Figure 1) (38, 39). Participants with T1D and T2D were recruited at the University Hospital Arnau Vilanova in Lleida and University Hospital Germans Trias i Pujol in Badalona (38, 39).

### 2.3. Eligibility criteria

#### 2.3.1. Inclusion and exclusion criteria

Individuals with a diagnosis of DR were included. For those with T1D, the criteria were being over 18 years of age with a duration of diabetes of at least 1 year. For those with T2D, individuals diagnosed with diabetes between the ages of 40 and 75 years were included.

Exclusion criteria were defined as being a healthcare professional, presenting with physical or cognitive impairments (e.g., dementia or mental health conditions), a history of clinical cardiovascular disease or diabetic foot disease, pregnancy, renal insufficiency (estimated glomerular filtration rate < 60 mL/min), and conditions requiring additional medical nutrition therapy, such as macroalbuminuria defined by a urine albumin-to-creatinine ratio >299 mg/g (38, 39). Both previous cohort studies were approved by the local Ethics Committee from University Hospital Arnau of Vilanova (CEIC 1079) (38, 39) and University Hospital Germans Trias i Pujol (PI-13-095 and PI-15-147) (40). Written informed consent was obtained from all the participants.

#### 2.3.2. Diagnosis of diabetic retinopathy

All participants from both cohorts underwent a comprehensive baseline examination by an ophthalmologist (41). For the ophthalmologic assessment, internationally standardized and validated criteria were used to classify and diagnose DR (42). The presence of different stages of DR was determined and classified into five stages according to the ETDRS (Early Treatment Diabetic Retinopathy Study) classification (43). (1) No retinopathy, (2) mild non-proliferative retinopathy (NPDR), (3) moderate NPDR, (4) severe NPDR, and (5) proliferative diabetic retinopathy (PRD) (44). For the analysis, subjects were grouped into three categories: (1) no retinopathy, (2) mild DR (ETDRS stage 2), and (3) advanced DR (ETDRS stages 3–5). Ophthalmological variables from the right eye were used if both eyes presented the same degree of DR; otherwise, variables from the eye with the highest degree of DR were used (45).

### 2.4. Other clinical data

Clinical and sociodemographic variables were collected from anamnesis, physical examination and information of medical records. Anthropometric variables (i.e., weight, body mass index [BMI] and waist circumference) were obtained by standardized methods (37, 38). Physical activity was evaluated using the validated method of Bernstein et al. (46) and Cabrera de León et al. (47). Hypertension and dyslipidemia were defined as receiving medication for these given conditions (i.e., antihypertension and lipid-lowering drugs, respectively). DM was classified according to the American Diabetes Association criteria (37, 38). Finally, blood samples were collected to determine biochemical measures using standard laboratory procedures.

### 2.5. Dietary niacin intake

Nutrient intake was evaluated by the validated 101-item FFQC, which was conducted through personal interviews by trained researchers (48). The FFQC measures the frequency of dietary intake, categorizing it into monthly, weekly, or daily consumption of various food groups, and collects data on food consumption over the year preceding the study visit (48). In addition, the FFQC has shown reproducibility for up to 5 years prior to the subject's visit (49). Nutrient intake data were derived from the U.S. Department of Agriculture composition tables, along with other food sources and serving sizes from both English and Spanish composition tables (50–52). Dietary Niacin and NEq intake of the 2 cohorts have been previously reported (36–38). Niacin and NEq (mg/day) intake values were obtained from the FFQC and adjusted for total energy intake; NEqs were calculated based on both tryptophan and niacin intake (48). The adjusted intake of niacin and NEq was assessed in relation to the Recommended Daily Allowance (RDA) for niacin and NEq, based on data from the European Food Safety Authority the RDA is set at 5.5 mg NE per 1,000 kcal per day (31).

### 2.6. Statistical methods

Statistical analyses were performed using the R project (version 3.3.2; https://www.r-project.org/). Categorical variables were presented as frequencies and percentages, which were reported as proportions at a 95% confidence interval (CI). Continuous variables were presented as mean and standard deviation (SD) or median with interquartile range (IQR), depending on their distribution. Differences between groups were analyzed with Mann–Whitney *U*-test, for continuous variables, depending on their distribution. For categorical variables, the chi-square test or Fisher's exact test was used, depending on the distribution of the variable. A minimum statistical significance threshold of *p* < 0.05 was applied to each comparison. The strength of association between the dependent variable (DR) and the independent variables [age, sex, systolic blood pressure (sBP), glycated hemoglobin (HbA1c), smoking, and BMI] were determined using logistic regression model analysis, and the results were reported as odds ratios (OR) at 95% confidence intervals. The multiple logistic regression was performed using three models. Only those variables that have been recognized as risk factors for DR were selected as confusing variables to adjust the logistic regression models applied in this study (37, 40, 53, 54). Model 1, adjusted for age and sex, Model 2, adjusted for age, sex and BMI, and sedentary physical activity, and Model 3, adjusted for the variables of model 2 plus smoking, hypertension, dyslipidemia and glomerular filtration rate (GFR).

A sensitivity analysis was conducted to explore the non-linear relationship between dietary niacin and NEq intake and DR was conducted using restricted cubic spline (RCS) regression with three knots (at the 10th, 50th, and 90th percentiles). A conservative type I error rate of 5% was used, with statistical significance defined as a *p* < 0.05. Is were analyzed to check for the null value (0 for categorical variables and 1 for continuous variables), ensuring the reliability of the results.

## 3 Results

### 3.1 Clinical and demographic data

A total of 534 participants were included of whom 45.5% had T1D and 54.5% had T2D. Among those with T1D, 42.3% had DR, while among participants with T2D, 49.5% had DR. The mean age of the T1D study group with DR was significantly higher (*p* = 0.004) compared with subjects without DR. Similarly, in the T2D group, the mean age of individuals with DR was higher (*p* = 0.024) that those without DR (Table 1).

**Table 1:** Sociodemographic, clinical, laboratory, and dietary intake variables of study participants.

**Characteristics**	**T1D**		***p*-value**	**T2D**		***p*-value**
	**No DR (*****n*** = **140)**	**DR (*****n*** = **103)**		**No DR (*****n*** = **147)**	**DR (*****n*** = **144)**	
**General characteristics**						
**Age, years**	**42.1** **±10.3**	**46.2** **±10.8**	**0.004** ^*^	**57.9** **±10.3**	**60.4** **±8.82**	**0.024** ^*^
Women, *n*, (%)	77.0 (55.0)	56.0 (54.4)	1.000	71.0 (48.3)	72 (50.0)	0.863
Smoking, *n*, (%)	67.0 (48.2)	55.0 (53.4)	0.503	81.0 (55.5)	71.0 (49.3)	0.350
Physical activity, sedentary, *n*, (%)	41.0 (30.1)	26.0 (25.2)	0.490	75.0 (51.0)	76.0 (52.8)	0.855
**Waist circumference, cm**	**87.0** **±12.1**	**90.7** **±13.4**	**0.033** ^*^	**104.0** **±12.1**	**107.0** **±11.1**	**0.034** ^*^
**sBP, mm Hg**	**123.0** **±16.1**	**131.0** **±18.4**	**0.001** ^*^	**134.0** **±15.5**	**145.0** **±19.9**	**< 0.001** ^*^
dBP, mm Hg	74.2 ± 9.6	74.0 ± 9.6	0.856	76.6 ± 10.5	77.0 ± 11.1	0.723
BMI, kg/m^2^	25.3 ± 4.0	26.2 ± 4.0	0.075	31.3 ± 5.12	31.9 ± 5.6	0.357
**Hypertension**, ***n*** **(%)**	**20.0 (14.3)**	**39.0 (37.9)**	**< 0.001** ^*^	**73.0 (49.7)**	**96.0 (66.7)**	**0.005** ^*^
Dyslipidemia, *n* (%)	48.0 (34.3)	48.0 (46.6)	0.071	64.0 (43.5)	74.0 (51.4)	0.221
**Diabetes duration, years**	**18.0** **±9.1**	**26.5** **±10.0**	**< 0.001** ^*^	**7.1** **±5.5**	**14.0** **±9.9**	**< 0.001** ^*^
eGFR	105.0 ± 13.8	102 ± 13.3	0.181	91.8 ± 14.4	89.2 ± 14.9	0.130
**Biochemistry**						
Glucose, mg/dl	161.0 (65.3)	168.0 (80.7)	0.424	**148.0 (48.7)**	**168.0 (60.6)**	**0.002** ^*^
**HbA1c, %**	**7.35** **±0.8**	**7.9** **±1.1**	**< 0.001** ^*^	**7.3** **±1.2**	**8.2** **±1.4**	**< 0.001** ^*^
**HbA1c, mmol/mol**	**56.9** **±8.4**	**62.9** **±11.9**	**< 0.001** ^*^	**55.9** **±12.7**	**66.6** **±15.6**	**< 0.001** ^*^
Total cholesterol, mg/dl	182.0 ± 27.0	179.0 ± 30.8	0.494	186.0 ± 36.7	185.0 ± 36.2	0.790
**HDL cholesterol, mg/dl**	**66.8** **±14.9**	**61.9** **±16.8**	**0.018** ^*^	**48.5** **±10.8**	**51.8** **±14.8**	**0.031** ^*^
LDL cholesterol, mg/dl	102.0 ± 22.7	102.0 ± 25.5	0.995	111.0 ± 30.8	106.0 ± 30.3	0.177
**Triglycerides, mg/dl**	**68.7** **±26.7**	**81.4** **±49.9**	**0.020** ^*^	138.0 ± 82.1	141.0 ± 121.0	0.827
**ALT, U/L**	**17.5** **±8.0**	**20.6** **±10.3**	**0.013** ^*^	28.3 ± 29.5	25.4 ± 18.2	0.303
AST, U/L	21.3 ± 7.6	22.0 ± 7.8	0.659	23.4 ± 16.8	22.9 ± 12.6	0.772
**Dietary niacin intake**						
Niacin, mg/day	27.7 ± 6.26	27.9 ± 5.99	0.813	29.2 ± 6.25	29.3 ± 8.05	0.861
NEq, mg/day	43.5 ± 7.78	43.9 ± 8.00	0.675	45.9 ± 8.40	46.8 ± 11.5	0.473

Data are mean (SD) for continuous variables and number (%) for categorical variables. ALT, alanine aminotransferase; AST, aspartate aminotransferase; BMI, body mass index; CKD, chronic kidney disease; dBP, diastolic blood pressure; DR, diabetic retinopathy; GFR: glomerular filtrate rate; HbA1c, glycosylated hemoglobin; HDL, high-density lipoprotein; LDL, low-density lipoprotein; NEq, niacin equivalents; sBP, systolic blood pressure; T1D, type 1 diabetes mellitus; T2D, type 2 diabetes mellitus. Niacin and NEq were adjusted for total daily caloric intake (kcal/day).

Participants with DR from T1D and T2D groups showed higher values of waist circumference (*p* = 0.033 and *p* = 0.034, respectively), systolic blood pressure (sBP) (*p* = 0.001 and *p* < 0.001, respectively), and HbA1c (*p* < 0.001 and *p* < 0.001, respectively). Additionally, participants with DR belonging to the T1D group exhibited lower levels of HDL cholesterol (*p* = 0.018), while those with DR and T2D showed higher levels (*p* = 0.031) of HDL cholesterol (Table 1).

In both study groups, T1D and T2D, participants with DR exhibited a higher frequency of hypertension (*p* < 0.001 and *p* = 0.005, respectively), and longer duration of DM (*p* < 0.001 and *p* < 0.001, respectively) than those without DR (Table 1).

Regarding dietary intake, no differences in niacin and NEq intake were seen across the T1D and T2D groups, regardless of DR status (Table 1). Similarly, no differences in niacin and NEq intake were found across the different DR categories of severity in either cohort (Supplementary Table 1).

### 3.2 Association analysis between dietary niacin and diabetic retinopathy

Subjects with DR in T1D and T2D groups did not exhibit differences in dietary niacin intake, analyzed as a categorical variable, compared with those without DR (Table 2). Similar results were obtained for dietary NEq intakes categorized by tertiles (Supplementary Table 2). Likewise, no differences were found in dietary intake of niacin and NEq, categorized by tertiles, when analyzed according to DR severity (Supplementary Tables 3, 4).

**Table 2:** Distribution of dietary niacin intake (mg/day) into tertiles in type 1 and type 2 diabetes mellitus groups distributed by tertiles.

**Niacin intake (mg/day)**								
	**T1D**				**T2D**			
	Tertile 1 (< 25) (*n* = 83)	Tertile 2 (25–30) (*n* = 84)	Tertile 3 (>30) (*n* = 76)	*p*-value	Tertile 1 (< 26) (*n* = 99)	Tertile 2 (26-31) (*n* = 91)	Tertile 3 (>31) (*n* = 101)	*p*-value
**Retinopathy**						
No DR	48 (57.8)	51 (60.7)	41 (53.9)	0.687	50 (50.5)	50 (54.9)	47 (46.5)	0.508
Yes DR	35 (42.2)	33 (39.3)	35 (46.1)		49 (49.5)	41 (45.1)	54 (53.5)	

DR, diabetic retinopathy; T1D, type 1 diabetes mellitus; T2D, type 2 diabetes mellitus.

Data are shown as number (%). The RCS regression analysis showed no association between dietary niacin intake and DR risk in individuals with T1D (*p* = 0.671). The regression curve showed log odds ratio (log OR) close to 0, indicating no clear association trends (Supplementary Figure 2). Neither significant association was found between dietary NEq and RD (*p* = 0.813) (Supplementary Figure 3). NEq intake appeared similar across all individuals with T1D, with no specific pattern related to incident DR.

The RCS regression analysis showed no association between dietary niacin intake and DR (*p* = 0.315) (Supplementary Figure 4). The log OR for DR in the subjects with T2D showed a plain U-shaped curve across the dietary niacin intake range. In this cohort, a similar picture was captured when analyzing the association between NEq intake and DR (*p* = 0.344) (Supplementary Figure 5).

### 3.3 Association analysis between dietary niacin, niacin equivalents and diabetic retinopathy in type 1 diabetes mellitus

**Figure 1:**
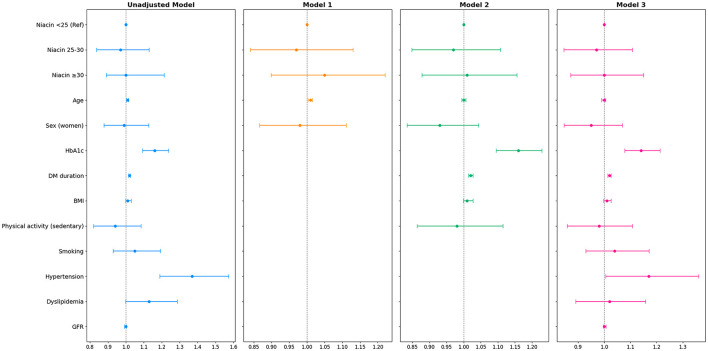
Forest plot of multiple logistic regression of dietary niacin intake (mg/day) in the type 1 diabetes mellitus group with diabetic retinopathy.

Dietary niacin intake showed no association with the presence of DR in participants with T1D. The unadjusted model indicated that age negatively influenced DR [OR (95% CI) = 1.01 (1.003, 1.015), *p* < 0.003], as did HbA1c levels and DM duration [OR (95% CI) = 1.16 (1.092, 1.238), *p* < 0.001]. Model 1 demonstrated a similar influence for age on DR [OR (95% CI) = 1.01 (1.003, 1.015), *p* = 0.003]. However, in Models 2 and 3, age did not retain its role as risk factor for DR. In contrast, both HbA1c and DM duration showed OR >1, suggesting an increased likelihood of DR in the presence of these two variables in the adjusted association models 2 and 3 (Figure 1; Supplementary Table 5). Similarly, niacin intake showed no association with the presence of DR when analyzed according to the different degrees of DR severity (Supplementary Tables 6–8).

The odds ratios (ORs) with their 95% confidence intervals (CIs) are shown. Model 1: adjusted for age and sex. Model 2: adjusted for age, sex, plus HbA1c, diabetes mellitus duration, BMI and sedentary physical activity. Model 3: adjusted for the variables of model 2 plus smoking, hypertension, dyslipidemia and GFR. The blue lines show the unadjusted model, while the orange, green, and magenta lines represent models 1, 2, and 3, respectively. The association between DR and niacin intake was calculated considering the relative effect measure of odds ratios (ORs) and the 95% confidence interval (CI). BMI, body mass index; DM, diabetes mellitus; GFR, glomerular filtration rate; HbA1c, glycosylated hemoglobin.

Dietary NEq intake showed no association with DR in the T1D group. Age was identified as a negative factor for DR, but only in the unadjusted model [OR (95% CI) = 1.01 (1.003, 1.015), *p* = 0.003] and in Model 1 [OR (95% CI) = 1.01 (1.003, 1.015), *p* = 0.003]. Higher HbA1c levels and longer DM duration were associated with an increased risk for DR in the unadjusted model, and after adjusting for sex and age (Model 2), and for all potential confounders (Model 3). Additionally, hypertension was a significant risk factor for DR in the unadjusted model [OR (95% CI) = 1.37 (1.189, 1.573), *p* < 0.001] that persisted after adjusting for all confounding variables (Model 3) [OR (95% CI) = 1.17 (1.003, 1.358), *p* = 0.047] (Supplementary Figure 6, Supplementary Table 9). Moreover, dietary NEq intake showed no association with DR in the T1D group when analyzed according to different degrees of DR severity (Supplementary Tables 10-12).

### 3.4 Association analysis between dietary niacin, niacin equivalents and diabetic retinopathy in type 2 diabetes

**Figure 2:**
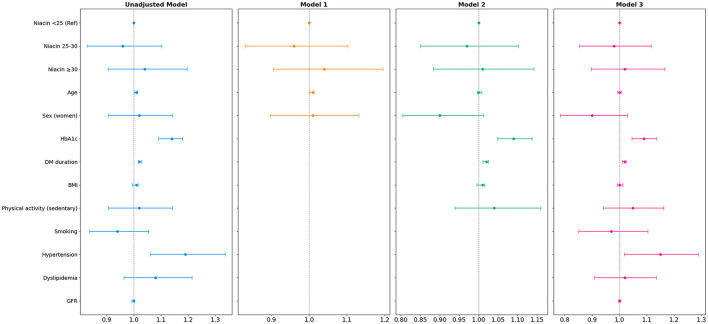
Forest plot of multiple logistic regression of niacin intake (mg/day) in type 2 diabetes group with diabetic retinopathy.

In the group of participants with T2D, dietary niacin intake showed no association with the presence of DR in any of the models (Figure 1, Supplementary Table 13). The unadjusted model indicated that age was an unfavorable factor for the development of DR [OR (95% CI) = 1.01 (1.001, 1.013), *p* = 0.02] as were HbA1c [OR (95% CI) = 1.14 (1.091, 1.180), *p* < 0.001] and the duration of T2D [OR (95% CI) = 1.02 (1.017, 1.029), *p* < 0.005]. Additionally, hypertension also was a risk factor for DR [OR (95% CI) = 1.19 (1.061, 1.336), *p* = 0.003]. In Model 1, the variable age exhibited the same impact as observed in the previous unadjusted model [OR (95% CI) = 1.01 (1.001, 1.013), *p* = 0.02], but did not retain its role as risk factor for DR in fully adjusted models. However, the negative impact of HbA1c and the duration of T2D on DR remained similar to that in the unadjusted model and Model 1 [OR (95% CI) = 1.09 (1.049, 1.137) *p* < 0.002] and [OR (95% CI) = 1.02 (1.011, 1.026), *p* < 0.001] (Figure 2; Supplementary Table 13). Higher HbA1c, longer DM duration, and hypertension remained deleterious factors for DR in the fully adjusted model 3 [OR (95% CI) = 1.09 (1.046, 1.136), *p* < 0.005, OR (95% CI) = 1.02 (1.011, 1.025), *p* < 0.005] and OR [95% CI = 1.19 (1.061, 1.336), *p* = 0.003, respectively] (Figure 2; Supplementary Table 13). Also, when the analysis was conducted according to DR severity, niacin intake showed no association with the presence of DR in T2D (Supplementary Tables 14-16).

The odds ratios (ORs) with their 95% confidence intervals (CIs) are shown. Model 1: adjusted for age and sex. Model 2: adjusted for age, sex, plus HbA1c, DM duration, BMI and sedentary physical activity. Model 3: adjusted for the variables of model 2 plus smoking, hypertension, dyslipidemia and GFR. The blue lines show the unadjusted model, while the orange, green, and magenta lines represent Models 1, 2, and 3, respectively. The association between DR and niacin intake was calculated with the relative effect measure of odds ratios (ORs) and the 95% confidence interval (CI). BMI, body mass index; DM, diabetes mellitus; GFR, glomerular filtration rate; HbA1c, glycosylated hemoglobin.

Dietary NEq intake was not associated with DR in the T2D group across all models. Age exhibited a non-beneficial association with DR only in the unadjusted model and Model 1 [OR (95% CI) = 1.00 (1.003, 1.015), *p* = 0.003] and [OR (95% CI) = 1.01 (1.003, 1.015), *p* = 0.002] (Supplementary Figure 7, Supplementary Table 17). Higher HbA1c levels and longer DM duration were both positively associated with RD, in the unadjusted model [OR (95% CI) = 1.16 (1.092, 1.238), *p* < 0.005] and [OR (95% CI) = 1.02 (1.014, 1.025), *p* < 0.001], as well as in Model 2 [OR (95% CI) = 1.16 (1.096, 1.230), *p* < 0.005] and [OR (95% CI) = 1.02 (1.014, 1.026), *p* < 0.001] and Model 3 [OR (95% CI) = 1.15 (1.079, 1.215), *p* < 0.005] and [OR (95% CI) = 1.02 (1.013, 1.026), *p* < 0.001]. Additionally, hypertension was observed to be associated with the presence of RD in both the unadjusted model and the fully adjusted Model 3 [OR (95% CI) = 1.37 (1.189, 1.573), *p* < 0.005] and [OR (95% CI) = 1.16 (1.003, 1.358), *p* = 0.04] (Supplementary Table 17, Supplementary Figure 7). In addition, dietary intake of NEq showed no association with DR in the T2D group when analyzed according to the different degrees of DR severity (Supplementary Tables 18-20).

## 4 Discussion

Despite accumulating evidence suggests that interventions with niacin or some of its derivatives may be protective against progression of vascular eye damage, the potential contribution of dietary niacin intake has not been ever assessed. Our association analysis revealed for the first time that dietary niacin consumption was not associated with DR in any of our cohorts. Noteworthy, and as previously described in subjects with T1D (40), the logistic regression analysis also confirmed the role of traditional diabetes-related variables, i.e., DM duration and higher HbA1c as the main contributors to DR incidence, in both the T1D and T2D groups. Our results also reinforced the well-established role of hypertension in DR progression (40, 54, 55).

In recent years, NAD+ depletion has been explored as a therapeutic target in DM (56, 57). Nicotinamide, a niacin derivative and NAD+ precursor, delayed T1D onset in experimental mouse models (58), but its benefits were not confirmed in large clinical trials like ENDIT and DENIS (59–61). Experimental studies suggest that oral nicotinamide mononucleotide improves cerebral microvascular circulation and neurovascular responses in mice (62), hinting at potential benefits for diabetic microangiopathy. In support of this, although unrelated to DM, other NAD+ precursors have increased intracellular NAD+ availability and have shown neuroprotective effects in retinal disease models (18–23), retinal vascular remodeling (25), and other neurodegenerative disorders (27, 28). Consistently, niacin supplementation has shown a neuroprotective effect in diabetic retinal neurodegeneration in an experimental model using male Sprague Dawley rats with DM by modulating oxidative stress (24). In human studies, the positive influence of niacin administration, or its derivatives, has been primarily reported in subjects with ocular conditions other than DR, including glaucoma (63–65), age-related macular degeneration (66), Graves' eye disease (67) and cataracts (68). Noteworthy, evidence from a small study suggest that niacin-induced vasodilation may accelerate retinal vascular occlusion resolution, while its discontinuation was associated with subnormal visual acuity, highlighting a potential therapeutic role in retinal vascular conditions (33). However, its clinical benefits in experimental or clinical diabetic retinal complications, such as DR, remain elusive. In the context of diabetic retinal disease, retinal vein occlusion, which has been reported to coexists with DR, has been reported to respond positively to niacin supplementation (69). Remarkably, all the above-mentioned studies are interventional, which essentially differ from our study approach, as we considered the analysis of the impact of dietary niacin consumption and therefore much closer to a real practice setting. Noteworthy, the impact of dietary niacin intake, rather than supplementation or pharmacological interventions on the incidence of DR in subjects with DM has not been assessed previously.

Our association models confirmed in both T1D and T2D groups that the presence of DR was closely related to main traditional risk factors, such as prolonged DM duration, poor glycemic control and also hypertension (53, 54, 70, 71). Consistently, both HbA1c levels and diabetes duration were negatively associated with DR in subjects with both T1D and T2D. Moreover, our adjusted analysis also revealed that other factors such as age and sex had any role in predicting incident DR in our cohorts.

To our knowledge, this is the first study to evaluate the association of dietary niacin and NEq intake with incident DR, considering them as dietary components rather than supplements. Another strength relies in the use of the FFQC, a tool with high reproducibility for assessing dietary intake, with data gathered by trained professionals, reflecting the participants' usual dietary intake. Ophthalmological examination used internationally standardized and validated criteria to classify and diagnose DR (42), thereby making our data to be comparable with other clinical and population-based studies. Nonetheless, this study has certain limitations. Its observational design restricts the ability to establish causal relationships between the analyzed variables. Additionally, DR is traditionally diagnosed and classified through an eye fundus examination, yet recent research suggests it represents the final stage of a prolonged process. Indeed, early DR may be initiated by hyperglycemia before retinal microangiopathic signs become visible. Furthermore, DR was analyzed both as a binary outcome (presence or absence) and classified into 3 severity stages (i.e., no retinopathy, mild DR and advanced DR), recognizing that greater severity generally reflects longer duration of DM (72–75), although data on duration of DR were not available. This may have limited the detection of specific associations, as the duration of DR could reflect irreversible microvascular damage that attenuates the effect of dietary factors such as niacin. Nevertheless, the lack of significant associations between niacin intake and the presence of DR may likely be influenced by unaccounted confounding factors.

## 5 Conclusion

Our study did not reveal a significant association between niacin or NEq intake and the presence of DR in subjects with T1D and T2D. The main determinants of DR in both groups were diabetes duration, HbA1c levels, and hypertension. Future studies should ideally include detailed staging and duration of DR to clarify whether nutrient-related effects vary across different stages of the disease. However, further research, particularly interventional studies, are needed to better understand the precise contribution of dietary niacin supplementation to DR.

## Data Availability

The raw data supporting the conclusions of this article will be made available by the authors, without undue reservation.
