# Supplementary material for: Association between dietary niacin intake and diabetic retinopathy in a Catalonian population: a cross-sectional study
Source: Front Nutr. 2025 Oct 14;12:1626379. doi: 10.3389/fnut.2025.1626379 (PMC12558777; doi:10.3389/fnut.2025.1626379)
Supplement: Supplementary file 1 [file Supplementary_file_1.docx]

Supplementary Material


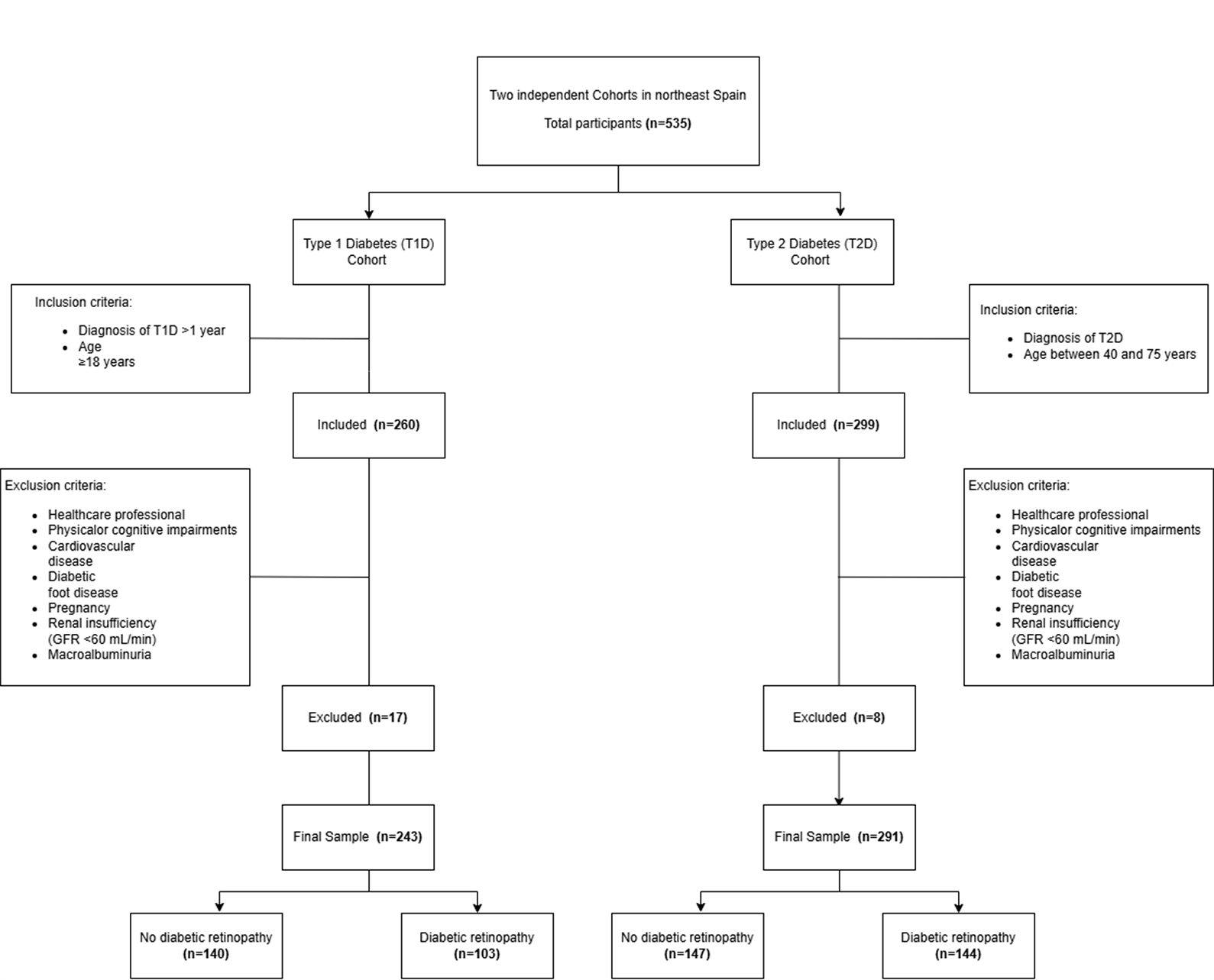


**Supplementary Figure 1.** Participant selection flowchart.

**Supplementary Table 1**. Sociodemographic, clinical, laboratory, and dietary intake variables of study participants distributed according to severity of retinopathy

| ***Variables*** | **T1D** | | | | **T2D** | | | |
| --- | --- | --- | --- | --- | --- | --- | --- | --- |
|  | **No DR**  **(n=140)** | **Mild DR**  **(n=67)** | **Advanced DR**  **(n=34)** | **p-value** | **No DR**  **(n=143)** | **Mild DR**  **(n=58)** | **Advanced DR**  **(n=83)** | **p-value** |
| *Clinical characteristics* | | | | | | | | |
| Age, years | 42.1 ± 10.3 | 45.4 ± 11 | 48.0 ± 10.2 | 0.006 | 57.9 ± 10.4 | 59.5 ± 8.95 | 61.4 ± 8.68 | 0.031 |
| Women, n, (%) | 77 (55.0) | 37 (55.2) | 18 (52.9) | 0.973 | 68 (47.6) | 25 (43.1) | 46 (55.4) | 0.317 |
| Smoking, n (%) | 67 (48.2) | 37 (55.2) | 17 (50) | 0.639 | 80 (56.3) | 31 (53.4) | 39 (47.0) | 0.398 |
| Physical activity, sedentary, n (%) | 41 (30.1) | 19 (28.4) | 7 (20.6) | 0.542 | 73 (51.0) | 30 (51.7) | 45 (54.2) | 0.898 |
| Waist circumference, cm | 87 ± 12.1 | 90.0 ± 11.5 | 92.3 ± 16.8 | 0.061 | 104 ± 11.7 | 105 ± 9.49 | 108 ± 11.8 | 0.025* |
| sBP, mm Hg | 123 ± 16.1 | 128 ± 15.2 | 137 ± 22.1 | <0.001* | 135 ± 15.1 | 139 ± 20.1 | 149 ± 19.1 | <0.001* |
| dBP, mm Hg | 74.2 ± 9.6 | 74.1 ± 8.4 | 74.3 ± 11.7 | 0.996 | 76.7 ± 10.4 | 76.6 ± 11.7 | 77.0 ± 10.6 | 0.975 |
| BMI, kg/m^2^ | 25.3 ± 4.0 | 26.0 ± 3.7 | 26.8 ± 5.3 | 0.100 | 31.1 ± 4.91 | 31.6 ± 5.33 | 32.0 ± 5.73 | 0.485 |
| Hypertension, n (%) | 20 (14.3) | 19 (28.4) | 20 (58.8) | <0.001* | 71 (49.7) | 37 (63.8) | 56 (67.5) | 0.019 |
| Dyslipidemia, n (%) | 48 (34.3) | 29 (43.3) | 18 (52.9) | 0.102 | 62 (43.4) | 33 (56.9) | 41 (49.4) | 0.208 |
| Diabetes duration, years | 18.0 ± 9.13 | 25.9 ± 10.3 | 28.1 ± 9.01 | <0.001* | 6.95 ± 5.49 | 12.5 ± 9.76 | 15.3 ± 10.0 | <0.001* |
| eGFR | 105 ± 13.8 | 104 ± 12.6 | 98.6 ± 14.4 | 0.058 | 91.9 ± 14.5 | 92.1 ± 13.1 | 86.7 ± 15.7 | 0.021 |
| *Biochemistry* | | | | | | | | |
| Glucose, mg/dL | 161 ± 65.3 | 175 ± 82.6 | 158 ± 78.0 | 0.344 | 147 ± 43.8 | 152 ± 52.1 | 178 ± 64.1 | <0.001* |
| HbA1c, % | 7.35 ± 0.76 | 7.70 ± 1.00 | 8.28 ± 1.17 | <0.001* | 7.25 ± 1.12 | 8.01 ± 1.45 | 8.39 ± 1.42 | <0.001* |
| HbA1c, mmol/mol | 56.9 ± 8.35 | 60.7 ± 10.9 | 66.9 ± 12.8 | <0.001* | 55.6 ± 12.3 | 64.1 ± 15.8 | 68.2 ± 15.5 | <0.001* |
| Total cholesterol, mg/dL | 182 ± 27.0 | 178 ± 28.2 | 180 ± 36.5 | 0.712 | 186 ± 36.9 | 189 ± 38.8 | 181 ± 34.3 | 0.475 |
| HDL cholesterol, mg/dL | 66.8 ± 14.9 | 62.1 ± 18.1 | 61.2 ± 13.7 | 0.049 | 48.5 ± 10.8 | 50.5 ± 15.0 | 53.1 ± 14.7 | 0.037* |
| LDL cholesterol, mg/dL | 102 ± 22.7 | 101 ± 22.8 | 105 ± 30.5 | 0.724 | 111 ± 30.9 | 108 ± 31.0 | 104 ± 29.7 | 0.232 |
| Triglycerides, mg/dL | 68.7 ± 26.7 | 80.8 ± 45.6 | 82.2 ± 58.8 | 0.045 | 138 ± 82.9 | 164 ± 170 | 126 ± 67.3 | 0.098 |
| ALT, U/L | 17.5 ± 8.00 | 20.8 ± 9.97 | 20.3 ± 11.3 | 0.031 | 28.5 ± 29.9 | 25.6 ± 14.7 | 23.4 ± 14.4 | 0.275 |
| AST, U/L | 21.3 ± 7.56 | 20.6 ± 5.97 | 24.4 ± 9.95 | 0.225 | 23.6 ± 17.0 | 22.6 ± 12.5 | 22.4 ± 11.7 | 0.828 |
| *Dietary niacin intake* | | | | | | | | |
| Niacin, mg/day | 27.7 ± 6.26 | 28.0 ± 5.77 | 27.7 ± 6.57 | 0.922 | 29.1 ± 6.23 | 29.5 ± 6.12 | 28.6 ± 6.68 | 0.706 |
| NEq, mg/day | 43.5 ± 7.78 | 44.3 ± 7.63 | 43.5 ± 8.84 | 0.767 | 45.9 ± 8.41 | 46.8 ± 8.35 | 45.8 ± 8.92 | 0.721 |

Data are mean (SD) for continuous variables and number (%) for categorical variables. ALT, alanine aminotransferase; AST, aspartate aminotransferase; BMI, body mass index; CKD, chronic kidney disease; dBP, diastolic blood pressure; DR, diabetic retinopathy; GFR: glomerular filtrate rate; HbA1c, glycosylated hemoglobin; HDL, high-density lipoprotein; LDL, low-density lipoprotein; sBP, systolic blood pressure; T1D, type 1 diabetes mellitus; T2D, type 2 diabetes mellitus. No DR, no diabetic retinopathy [1]; Mild DR [ETDRS stages 2]; advanced DR [ETDRS stages 3–5]. Niacin and NEq were adjusted for total daily caloric intake (kcal/day).

**Supplementary Table 2.** Dietary niacin equivalents intake (mg/day) in type 1 and type 2 diabetes mellitus groups distributed by tertiles.

| Niacin equivalents intake (mg/day) | | | | | | | | | | |
| --- | --- | --- | --- | --- | --- | --- | --- | --- | --- | --- |
|  | T1D | | | | | T2D | | | | |
|  | Tertile 1 (<40)  (n=82) | Tertile 2 (40-46)  (n=84) | Tertile 3 (>46)  (n=77) | *p*-value | Tertile 1 (<42)  (n=92) | | Tertile 2 (42-49)  (n=104) | Tertile 3 (>49)  (n=95) | *p*-value |  |
| No DR | 49 (59.8) | 50 (59.5) | 41 (53.2) | 0.644 | 49 (53.3) | | 59 (56.7) | 39 (41.1) | 0.071 |  |
| Yes DR | 33 (40.2) | 34 (40.5) | 36 (46.8) |  | 43 (46.7) | | 45 (43.3) | 56 (58.9) |  |  |

Data are shown as number (%). DR: diabetic retinopathy; T1D, type 1 diabetes mellitus; T2D, type 2 diabetes mellitus.

**Supplementary Table 3.** Dietary niacin intake (mg/day) in type 1 and type 2 diabetes mellitus groups distributed by tertiles according to severity of retinopathy.

| **Niacin intake (mg/day)** | | | | | | | | |
| --- | --- | --- | --- | --- | --- | --- | --- | --- |
|  | **T1D** | | | | **T2D** | | | |
|  | **Tertile 1 (<25)** | **Tertile 2 (25-30)** | **Tertile 3 (>30)** | **p-value** | **Tertile 1 (<26)** | **Tertile 2 (26-31)** | **Tertile 3 (>31)** | **p-value** |
|  | (n=82) | (n=83) | (n=76) |  | (n=96) | (n=90) | (n=98) |  |
| **RD grade** |  |  |  | 0.640 |  |  |  | 0.603 |
| **No RD** | 48 (58.5) | 51 (61.4) | 41 (53.9) |  | 49 (51.0) | 49 (54.4) | 45 (45.9) |  |
| **Mild RD** | 21 (25.6) | 20 (24.1) | 26 (34.2) |  | 18 (18.8) | 15 (16.7) | 25 (25.5) |  |
| **Advanced RD** | 13 (15.9) | 12 (14.5) | 9 (11.8) |  | 29 (30.2) | 26 (28.9) | 28 (28.6) |  |

Data are shown as number (%). DR, diabetic retinopathy; No DR, no diabetic retinopathy [1]; Mild DR [ETDRS stages 2]; advanced DR [ETDRS stages 3–5]; T1D, type 1 diabetes mellitus; T2D, type 2 diabetes mellitus.

**Supplementary Table 4.** Dietary niacin equivalents intake (mg/day) in type 1 and type 2 diabetes mellitus groups distributed by tertiles according to severity of retinopathy.

| **Niacin equivalents intake (mg/day)** | | | | | | | | |
| --- | --- | --- | --- | --- | --- | --- | --- | --- |
|  | **T1D** | | | | **T2D** | | | |
|  | **Tertile 1 (<40)** | **Tertile 2 (40-46)** | **Tertile 3 (>46)** | **p-value** | **Tertile 1 (<42)** | **Tertile 2 (42-49)** | **Tertile 3 (>49)** | **p-value** |
|  | (n=80) | (n=84) | (n=77) |  | (n=91) | (n=101) | (n=92) |  |
| **DR grade** |  |  |  | 0.644 |  |  |  | 0.104 |
| **No DR** | 49 (61.3) | 50 (59.5) | 41 (53.2) |  | 48 (52.7) | 58 (57.4) | 37 (40.2) |  |
| **Mild DR** | 17 (21.2) | 25 (29.8) | 25 (32.5) |  | 17 (18.7) | 15 (14.9) | 26 (28.3) |  |
| **Advanced DR** | 14 (17.5) | 9 (10.7) | 11 (14.3) |  | 26 (28.6) | 28 (27.7) | 29 (31.5) |  |

Data are shown as number (%). DR: diabetic retinopathy; No DR, no diabetic retinopathy [1]; Mild DR [ETDRS stages 2]; advanced DR [ETDRS stages 3–5]; T1D, type 1 diabetes mellitus; T2D, type 2 diabetes mellitus.


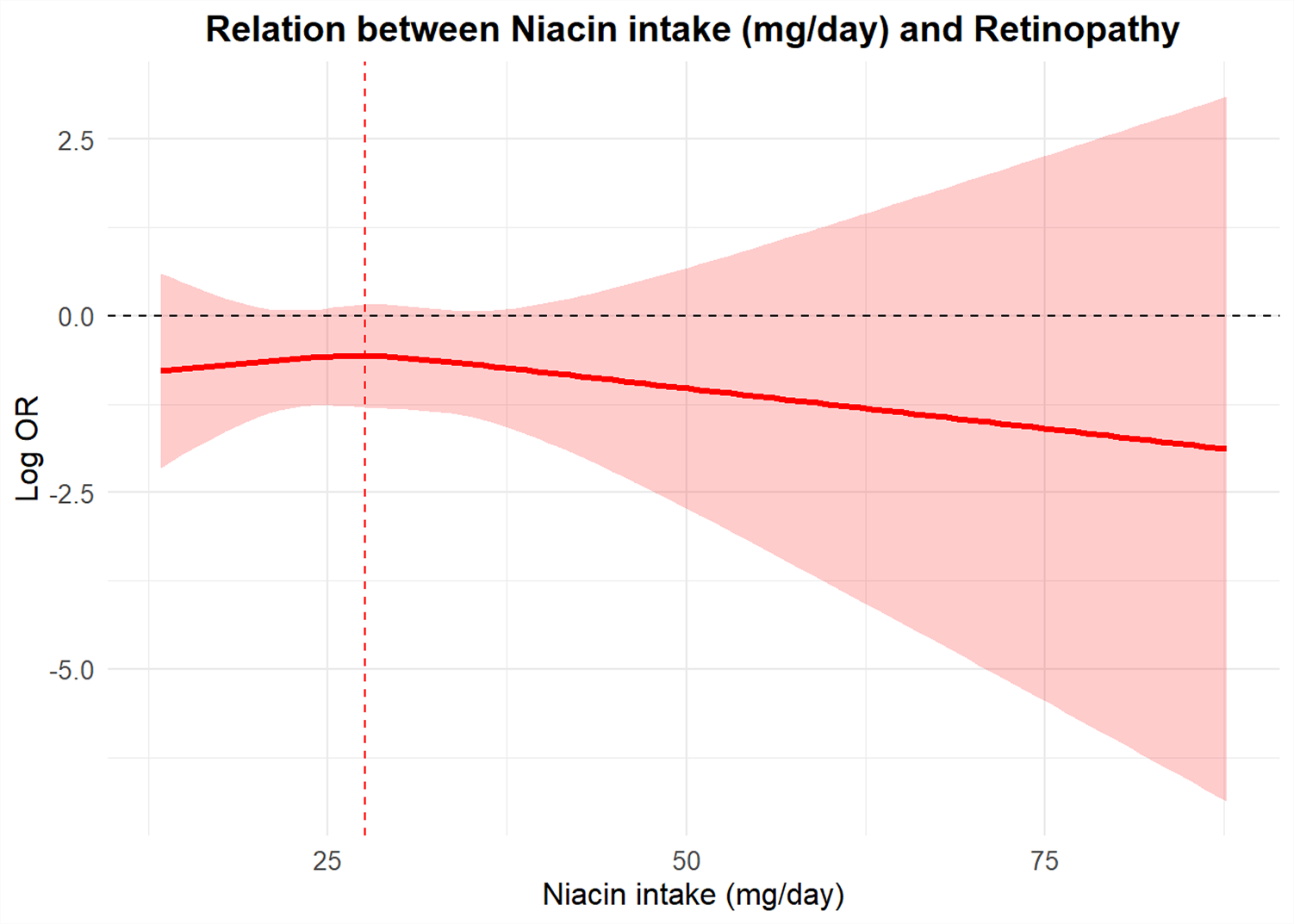


*p*-value for non-lineal trend = 0.671

**Supplementary Figure 2.** Restricted cubic spline regression analysis of the association between dietary niacin intake (mg/day) and diabetic retinopathy in type 1 diabetes mellitus group.

*p*-value for non-lineal trend = 0.671

Restricted cubic spline regression was adjusted for age, sex, HbA1c, diabetes duration, body mass index, sedentary physical activity, smoking, hypertension, dyslipidemia and glomerular filtrate rate. The X-axis shows niacin intake (mg/day) as a continuous variable, while the Y-axis displays the Log ORs (OR) for the likelihood of developing DR. The greyish red shading represents the confidence interval (CI). The dashed red lines indicate the trend in niacin intake among participants with T1D and DR. Abbreviations used were: DR, diabetic retinopathy; OR, odds ratio.


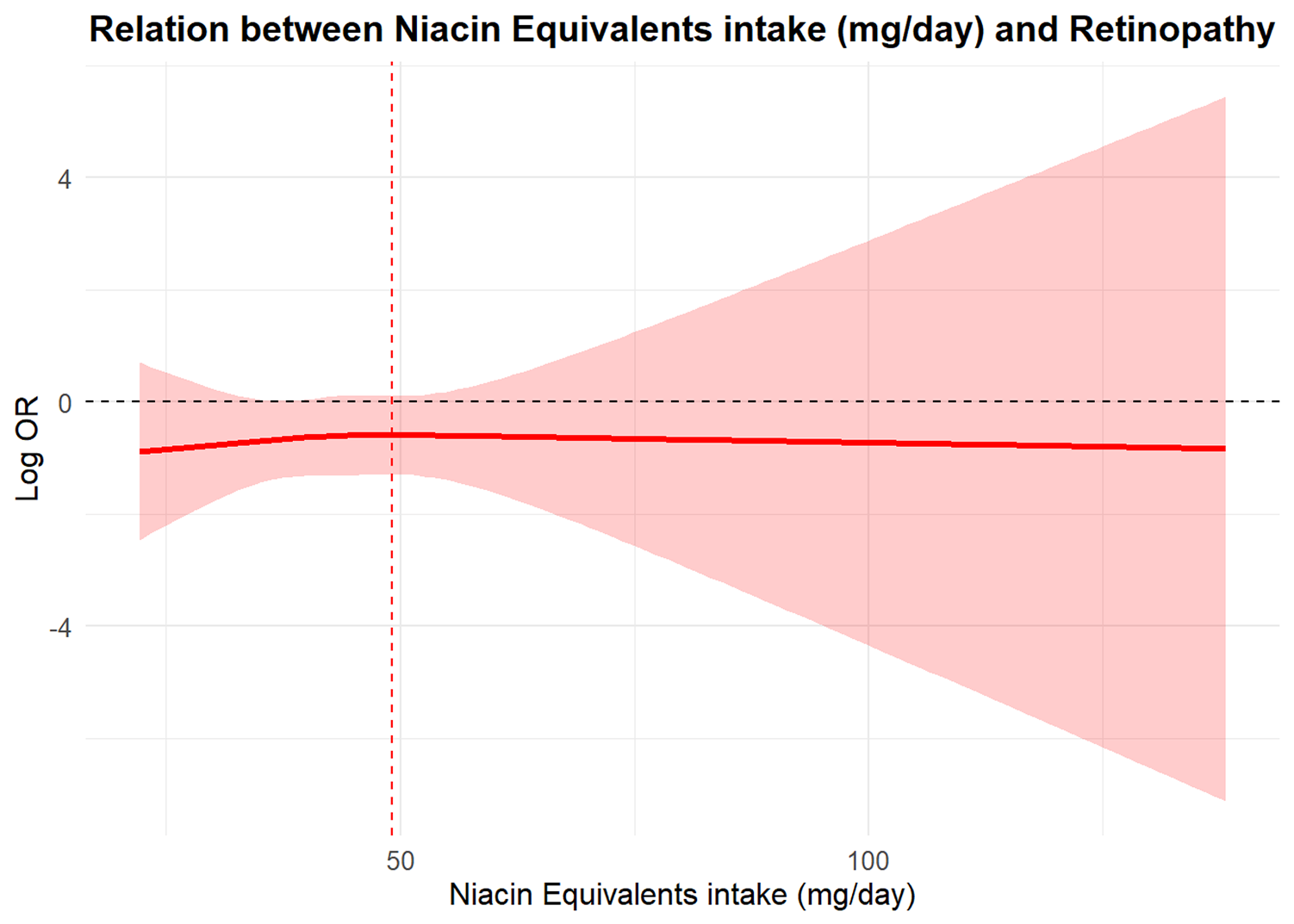


*p*-value for non lineal trend = 0.813

**Supplementary Figure 3.** Restricted cubic spline regression analysis of the association between dietary niacin equivalents intake (mg/day) and diabetic retinopathy in type 1 diabetes mellitus group.

*p*-value for non lineal trend = 0.813

Restricted cubic spline regression was adjusted for age, sex, HbA1c, diabetes mellitus duration, body mass index, sedentary physical activity, smoking, hypertension, dyslipidemia and glomerular filtrate rate. The X-axis shows niacin equivalents intake (mg/day) as a continuous variable, while the Y-axis displays the Log ORs (OR) for the likelihood of developing DR. The greyish red shading represents the confidence interval (CI). The dashed red lines indicate the trend in niacin intake among participants with T1D and DR. Abbreviations used were: DR, diabetic retinopathy; OD, odds ratio.


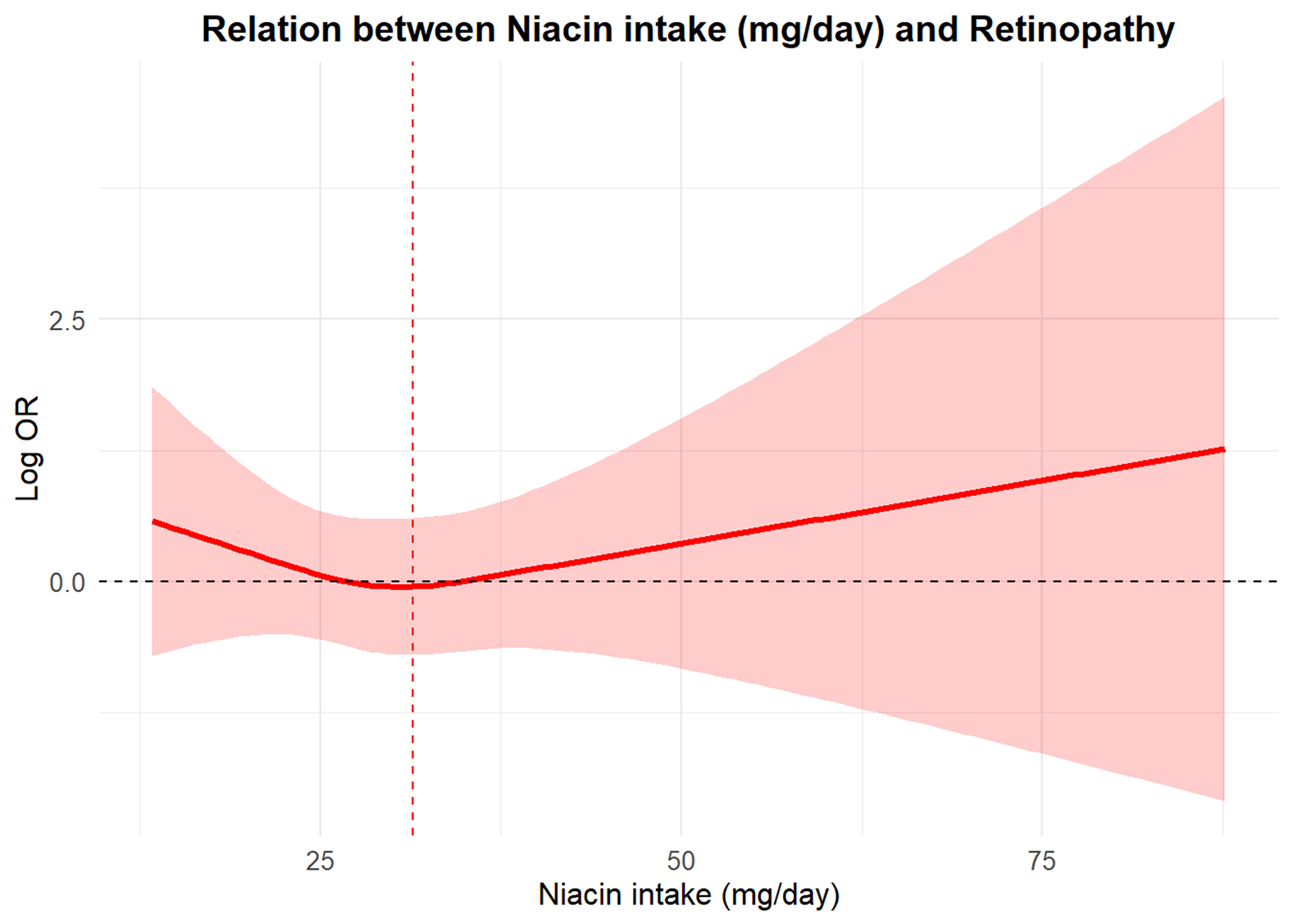


*p*-value for non-lineal trend= 0.315

**Supplementary Figure. 4.** Restricted cubic spline regression analysis of the association between dietary niacin intake (mg/day) and diabetic retinopathy in type 2 diabetes mellitus group.

*p*-value for non-lineal trend= 0.315

Restricted cubic spline regression was adjusted for age, sex, HbA1c, diabetes duration, body mass index, sedentary physical activity, smoking, hypertension, dyslipidemia and glomerular filtrate rate. The X-axis shows niacin intake (mg/day) as a continuous variable, while the Y-axis displays the Log ORs (OR) for the likelihood of developing DR. The greyish red shading represents the confidence interval (CI). The dashed red lines indicate the trend in niacin intake among participants with T2D and DR. Abbreviations used were: DR, diabetic retinopathy; OD, odds ratio.


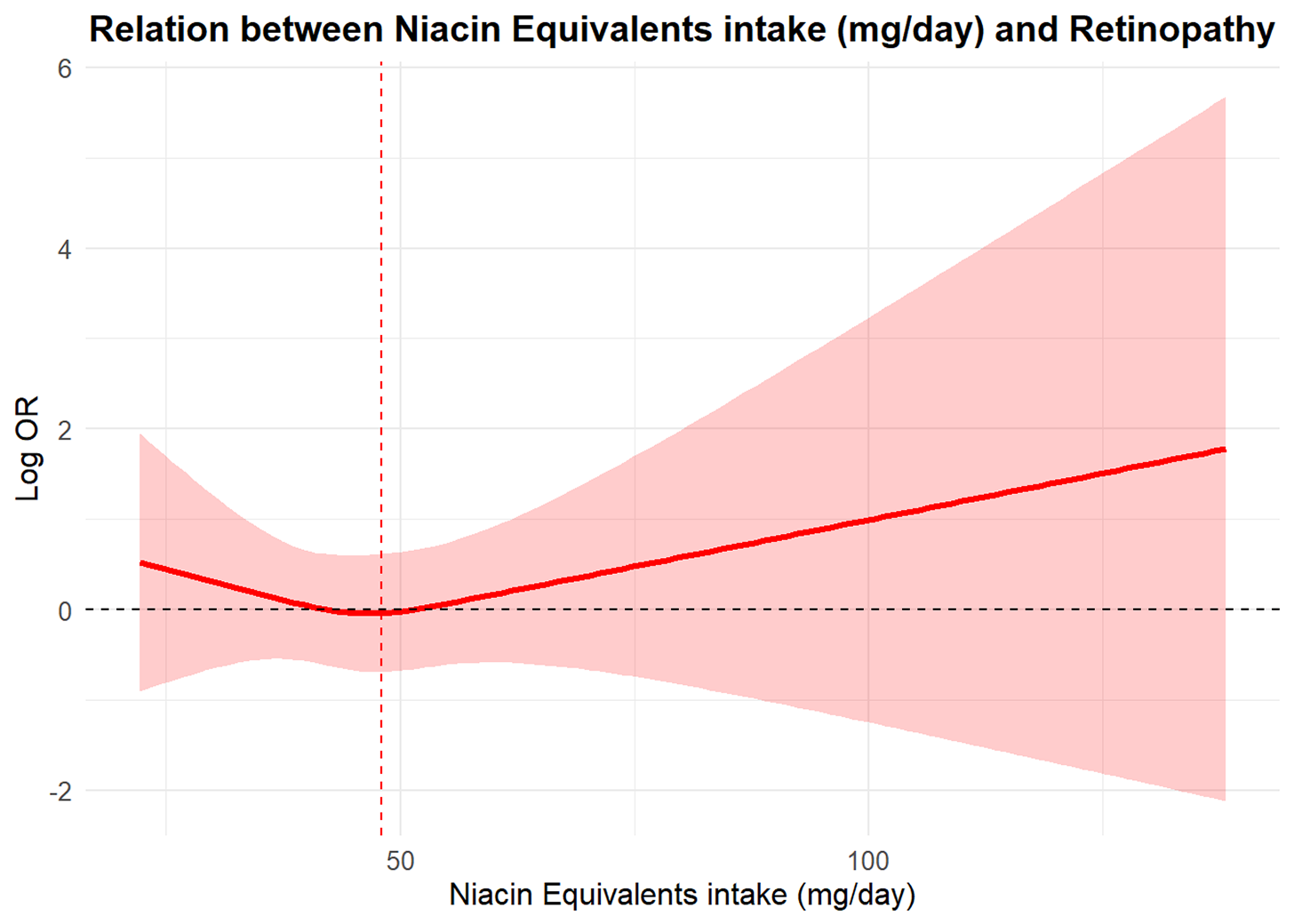


*p*-value for non lineal trend = 0.344

**Supplementary Figure 5.** Restricted cubic spline regression analysis of the association between dietary niacin equivalents intake (mg/day) and diabetic retinopathy in type 2 diabetes mellitus group.

*p*-value for non lineal trend = 0.344

Restricted cubic spline regression was adjusted for age, sex, HbA1c, diabetes mellitus duration, body mass index, sedentary physical activity, smoking, hypertension, dyslipidemia and glomerular filtrate rate. The X-axis shows niacin intake (mg/day) as a continuous variable, while the Y-axis displays the Log ORs (OR) for the likelihood of developing DR. The greyish red shading represents the confidence interval (CI). The dashed red lines indicate the trend in niacin intake among participants with T2D and DR. Abbreviations used were: DR, diabetic retinopathy; OD, odds ratio.

**Supplementary Table 5.** Multiple logistic regression models for association between niacin intake and diabetic retinopathy before and after adjusting for patient sex, age, clinical variables and comorbidities of type 1 diabetes mellitus.

| **Retinopathy** | | | | | | | | |
| --- | --- | --- | --- | --- | --- | --- | --- | --- |
|  | **Unadjusted model** |  | **Model 1** |  | **Model 2** |  | **Model 3** |  |
| Niacin intake (mg/day) | OR (CI) | *p*-value | OR (CI) | *p*-value |  | *p*-value |  | *p*-value |
| <25 (Ref) | 1.00 |  | 1.00 |  | 1.00 |  | 1.00 |  |
| 25-30 | 0.97 (0.836- 1.129) | 0.708 | 0.97 (0.840, 1.130) | 0.730 | 0.97 (0.848, 1.108) | 0.649 | 0.97 (0.845, 1.108) | 0.631 |
| ≥30 | 1.00 (0.891, 1.213) | 0.623 | 1.05 (0.899, 1.221) | 0.551 | 1.01 (0.878, 1.156) | 0.917 | 1.00 (0.872, 1.150) | 0.985 |
| Age | 1.01 (1.003, 1.015) | 0.003 | 1.01 (1.003, 1.015) | 0.003 | 1.00 (0.994, 1.006) | 0.929 | 1.00 (0.990, 1.005) | 0.564 |
| Sex (women) | 0.99 (0.877, 1.127) | 0.923 | 0.98 (0.866, 1.111) | 0.763 | 0.93 (0.834, 1.044) | 0.227 | 0.95 (0.847, 1.069) | 0.404 |
| HbA1c | 1.16 (1.092, 1.238) | <0.001 |  |  | 1.16 (1.095, 1.229) | <0.001 | 1.14 (1.078, 1.213) | <0.001* |
| DM duration | 1.02 (1.014, 1.025) | <0.001 |  |  | 1.02 (1.014, 1.027) | <0.001 | 1.02 (1.013, 1.026) | <0.001* |
| BMI | 1.01 (0.999, 1.029) | 0.072 |  |  | 1.01 (0.999, 1.027) | 0.072 | 1.01 (0.998, 1.026) | 0.100 |
| Physical activity (sedentary) | 0.94 (0.819, 1.084) | 0.405 |  |  | 0.98 (0.864, 1.115) | 0.774 | 0.98 (0.858, 1.108) | 0.698 |
| Smoking | 1.05 (0.929, 1.192) | 0.426 |  |  |  |  | 1.04 (0.929, 1.171) | 0.477 |
| Hypertension | 1.37 (1.189, 1.573) | 0.053 |  |  |  |  | 1.17 (1.005, 1.361) | 0.044* |
| Dyslipidemia | 1.13 (0.999, 1.287) | 0.183 |  |  |  |  | 1.02 (0.891, 1.157) | 0.820 |
| GFR | 1.00 (0.992, 1.001) |  |  |  |  |  | 1.00 (0.996, 1.007) | 0.588 |

The odds ratios (ORs) with their 95% confidence intervals (CIs) are shown. BMI, Body Mass Index; DM, Diabetes Mellitus; GFR, glomerular filtration rate; HbA1c, glycosylated hemoglobin; OR, odds ratio; CI, confidence interval. Model 1: adjusted for age and sex. Model 2: adjusted for age, sex, plus HbA1c, DM duration, BMI and sedentary physical activity. Model 3: adjusted for the variables of model 2 plus smoking, hypertension, dyslipidemia and GFR.

**Supplementary Table 6.** Multiple logistic regression models for association between niacin intake and mild diabetic retinopathy (No DR vs. Mild DR), before and after adjustment for sex, age, clinical variables, and comorbidities in type 1 diabetes mellitus.

| **Mild DR (T1D)** | | | | | | | | |
| --- | --- | --- | --- | --- | --- | --- | --- | --- |
|  | **Unadjusted model** |  | **Model 1** |  | **Model 2** |  | **Model 3** |  |
| **Niacin intake (mg/day)** | **OR (CI)** | **p-value** | **OR (CI)** | **p-value** |  | **p-value** |  | **p-value** |
| <25 (Ref) | 1.00 (ref) |  | 1.00 (ref) |  | 1.00 (ref) |  | 1.00 (ref) |  |
| 25-30 | 0.90 (0.433, 1.857) | 0.768 | 0.89 (0.428, 1.868) | 0.766 | 0.87 (0.375, 2.000) | 0.737 | 0.87 (0.369, 2.025) | 0.738 |
| ≥30 | 1.45 (0.712, 2.949) | 0.306 | 1.46 (0.710, 2.999) | 0.304 | 1.31 (0.586, 2.907) | 0.515 | 1.25 (0.554, 2.800) | 0.595 |
| **Age** | 1.03 (1.002, 1.060) | 0.039 | 1.03 (1.002, 1.061) | 0.038 | 1,00 (0.959, 1.031) | 0.768 | 0.99 (0.945, 1.039) | 0.705 |
| Sex (women) | 1.01 (0.562, 1.812) | 0.976 | 0.98 (0.538, 1.787) | 0.949 | 0.71 (0.363, 1.396) | 0.323 | 0.77 (0.385, 1.523) | 0.447 |
| HbA1c | 1.60 (1.130, 2.256) | 0.008 |  |  | 1.77 (1.209, 2.596) | 0.003* | 1.73 (1.171, 2.543) | 0.006* |
| DM duration | 1.09 (1.051, 1.126) | <0.001* |  |  | 1.10 (1.059, 1.150) | <0.001* | 1.10 (1.058, 1.150) | <0.001* |
| BMI | 1.05 (0.976, 1.132) | 0.190 |  |  | 1.08 (0.985, 1.173) | 0.106 | 1.07 (0.979, 1.169) | 0.139 |
| Physical activity (sedentary) | 0.92 (0.481, 1.749) | 0.793 |  |  | 1.00 (0.472, 2.117) | 1,000 | 0.97 (0.452, 2.094) | 0.944 |
| Smoking | 1.33 (0.738, 2.380) | 0.346 |  |  |  |  | 1.41 (0.716, 2.794) | 0.319 |
| Hypertension | 2.38 (1.166, 4.838) | 0.017* |  |  |  |  | 1.60 (0.647, 3.976) | 0.308 |
| Dyslipidemia | 1.46 (0.806, 2.655) | 0.211 |  |  |  |  | 1.07 (0.490, 2.327) | 0.870 |
| GFR | 1.00 (0.975, 1.018) | 0.717 |  |  |  |  | 1.01 (0.979, 1.041) | 0.530 |

The odds ratios (ORs) with their 95% confidence intervals (CIs) are shown. BMI, Body Mass Index; DM, Diabetes Mellitus; GFR, glomerular filtration rate; HbA1c, glycosylated hemoglobin; OR, odds ratio; CI, confidence interval. Model 1: adjusted for age and sex. Model 2: adjusted for age, sex, plus HbA1c, DM duration, BMI and sedentary physical activity. Model 3: adjusted for the variables of model 2 plus smoking, hypertension, dyslipidemia and GFR. No DR, no diabetic retinopathy [1]; Mild DR [ETDRS stages 2].

**Supplementary Table 7.** Multiple logistic regression models for association between niacin intake and advanced diabetic retinopathy (No DR vs. Advanced DR), before and after adjustment for sex, age, clinical variables, and comorbidities in type 1 diabetes mellitus.

| **Advanced DR (T1D)** | | | | | | | | |
| --- | --- | --- | --- | --- | --- | --- | --- | --- |
|  | **Unadjusted model** |  | **Model 1** |  | **Model 2** |  | **Model 3** |  |
| **Niacin intake (mg/day)** | **OR (CI)** | **p-value** | **OR (CI)** | **p-value** |  | **p-value** |  | **p-value** |
| <25 (Ref) | 1.00 (ref) |  | 1.00 (ref) |  | 1.00 (ref) |  | 1.00 (ref) |  |
| 25-30 | 0.87 (0.361, 2.090) | 0.754 | 0.96 (0.387, 2.400) | 0.9361 | 1.03 (0.318, 3.319) | 0.963 | 0.80 (0.232, 2.774) | 0.729 |
| ≥30 | 0.81 (0.315, 2.089) | 0.664 | 0.84 (0.311, 2.251) | 0.7245 | 0.78 (0.219, 2.752) | 0.695 | 0.66 (0.177, 2.477) | 0.541 |
| Age | 1.06 (1.017, 1.097) | 0.005 | 1.06 (1.017, 1.097) | 0.005 | 1.02 (0.964, 1.078) | 0.493 | 0.98 (0.911, 1.051) | 0.556 |
| Sex (women) | 0.92 (0.434, 1.951) | 0.829 | 0.88 (0.402, 1.934) | 0.753 | 0.70 (0.257, 1.920) | 0.492 | 0.77 (0.260, 2.300) | 0.643 |
| HbA1c | 2.82 (1.783, 4.459) | <0.001* |  |  | 3.76 (2.150, 6.569) | <0.001* | 3.42 (1.896, 6.168) | <0.001* |
| DM duration | 1.11 (1.064, 1.162) | <0.001* |  |  | 1.13 (1.068, 1.200) | <0.001* | 1.13 (1.061, 1.201) | <0.001* |
| BMI | 1.09 (0.998, 1.180) | 0.057 |  |  | 1.12 (1.002, 1.251) | 0.045 | 1.12 (0.990, 1.258) | 0.073 |
| Physical activity (sedentary) | 0.60 (0.242, 1.490) | 0.272 |  |  | 0.62 (0.179, 2.150) | 0.452 | 0.53 (0.128, 2.145) | 0.369 |
| Smoking | 1.08 (0.508, 2.275) | 0.851 |  |  |  |  | 1.12 (0.375, 3.359) | 0.837 |
| Hypertension | 8.57 (3.734, 19.677) | <0.001* |  |  |  |  | 5.24 (1.635, 16.757) | 0.005* |
| Dyslipidemia | 2.16 (1.010, 4.604) | 0.047 |  |  |  |  | 1.11 (0.345, 3.583) | 0.859 |
| GFR | 0.97 (0.945, 0.996) | 0.024 |  |  |  |  | 1.00 (0.956, 1.040) | 0.897 |

The odds ratios (ORs) with their 95% confidence intervals (CIs) are shown. BMI, Body Mass Index; DM, Diabetes Mellitus; GFR, glomerular filtration rate; HbA1c, glycosylated hemoglobin; OR, odds ratio; CI, confidence interval. Model 1: adjusted for age and sex. Model 2: adjusted for age, sex, plus HbA1c, DM duration, BMI and sedentary physical activity. Model 3: adjusted for the variables of model 2 plus smoking, hypertension, dyslipidemia and GFR. No DR, no diabetic retinopathy [1]; advanced DR [ETDRS stages 3–5].

**Supplementary Table 8.** Multiple logistic regression models for association between niacin intake and diabetic retinopathy (Mild DR vs. Advanced DR), before and after adjustment for sex, age, clinical variables, and comorbidities in type 1 diabetes mellitus.

| **Mild DR vs Advanced DR (T1D)** | | | | | | | | |
| --- | --- | --- | --- | --- | --- | --- | --- | --- |
|  | **Unadjusted model** |  | **Model 1** |  | **Model 2** |  | **Model 3** |  |
| **Niacin intake (mg/day)** | **OR (CI)** | **p-value** | **OR (CI)** | **p-value** |  | **p-value** |  | **p-value** |
| <25 (Ref) | 1.00 (ref) |  | 1.00 (ref) |  | 1.00 (ref) |  | 1.00 (ref) |  |
| 25-30 | 0.97 (0.358, 2.622) | 0.951 | 0.94 (0.345, 2.576) | 0.9081 | 0.99 (0.334, 2.915) | 0.981 | 0.95 (0.301, 3.007) | 0.932 |
| ≥30 | 0.56 (0.200, 1.560) | 0.267 | 0.61 (0.215, 1.756) | 0.3628 | 0.59 (0.185, 1.852) | 0.362 | 0.57 (0.167, 1.952) | 0.372 |
| Age | 1.02 (0.984, 1.063) | 0.263 | 1.02 (0.978, 1.061) | 0.3709 | 1.01 (0.962, 1.060) | 0.696 | 0.98 (0.920, 1.040) | 0.484 |
| Sex (women) | 0.91 (0.399, 2.087) | 0.828 | 0.86 (0.366, 2.033) | 0.7362 | 0.54 (0.204, 1.449) | 0.223 | 0.53 (0.176, 1.598) | 0.259 |
| HbA1c | 1.63 (1.096, 2.419) | 0.016 |  |  | 2.09 (1.306, 3.357) | 0.002* | 2.02 (1.244, 3.289) | 0.004* |
| DM duration | 1.02 (0.982, 1.068) | 0.271 |  |  | 1.05 (0.992, 1.105) | 0.094 | 1.04 (0.986, 1.104) | 0.142 |
| BMI | 1.05 (0.950, 1.151) | 0.361 |  |  | 1.08 (0.965, 1.203) | 0.186 | 1.06 (0.938, 1.198) | 0.349 |
| Physical activity (sedentary) | 0.66 (0.244, 1.757) | 0.401 |  |  | 0.59 (0.194, 1.795) | 0.353 | 0.60 (0.188, 1.917) | 0.389 |
| Smoking | 0.81 (0.355, 1.854) | 0.619 |  |  |  |  | 0.61 (0.208, 1.788) | 0.368 |
| Hypertension | 3.61 (1.519, 8.574) | 0.004* |  |  |  |  | 3.59 (1.122, 11.483) | 0.031* |
| Dyslipidemia | 1.47 (0.644, 3.377) | 0.359 |  |  |  |  | 0.78 (0.251, 2.394) | 0.657 |
| GFR | 0.97 (0.938, 1.001) | 0.055 |  |  |  |  | 0.99 (0.944, 1.033) | 0.587 |

The odds ratios (ORs) with their 95% confidence intervals (CIs) are shown. BMI, Body Mass Index; DM, Diabetes Mellitus; GFR, glomerular filtration rate; HbA1c, glycosylated hemoglobin; OR, odds ratio; CI, confidence interval. Model 1: adjusted for age and sex. Model 2: adjusted for age, sex, plus HbA1c, DM duration, BMI and sedentary physical activity. Model 3: adjusted for the variables of model 2 plus smoking, hypertension, dyslipidemia and GFR. Mild DR [ETDRS stages 2]; advanced DR [ETDRS stages 3–5].


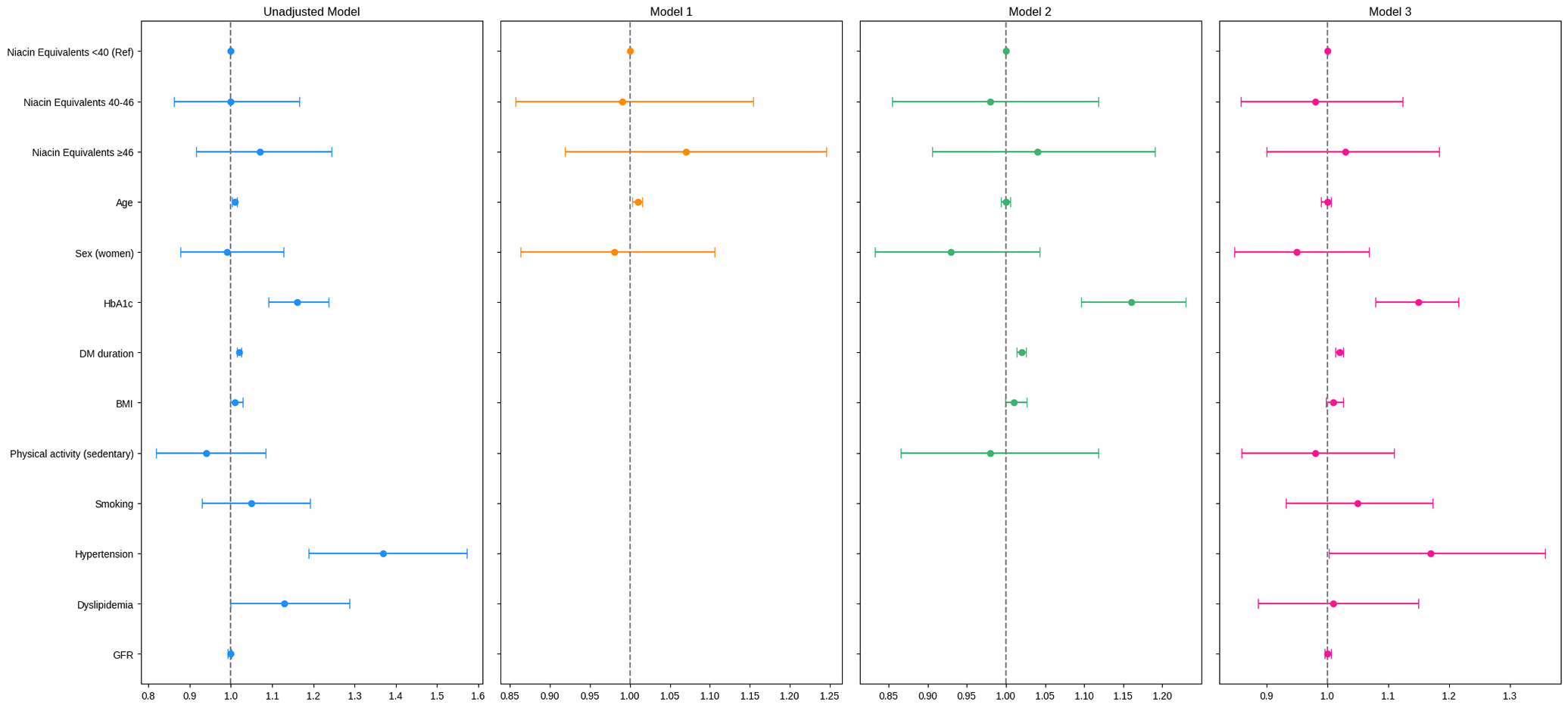


**Supplementary Figure 6.** Forest plot of multiple logistic regression of dietary niacin equivalents intake (mg/day) in type 1 diabetes mellitus group with diabetic retinopathy

The odds ratios (ORs) with their 95% confidence intervals (CIs) are represented. BMI, Body Mass Index; DM, Diabetes Mellitus; GFR, glomerular filtration rate; HbA1c, glycosylated hemoglobin. Model 1: adjusted for age and sex. Model 2: adjusted for age, sex, plus HbA1c, DM duration, BMI and sedentary physical activity. Model 3: adjusted for the variables of model 2 plus smoking, hypertension, dyslipidemia and GFR.

**Supplementary Table 9.** Multiple logistic regression models for association between dietary niacin equivalents intake and diabetic retinopathy in type 1 diabetes mellitus group before and after adjusting for patient sex, age, clinical variables and comorbidities.

| **Retinopathy** | | | | | | | | |
| --- | --- | --- | --- | --- | --- | --- | --- | --- |
|  | **Unadjusted model** |  | **Model 1** |  | **Model 2** |  | **Model 3** |  |
| Niacin equivalents intake (mg/day) | OR (CI) | *p*-value | OR (CI) | *p*-value |  | *p*-value |  | *p*-value |
| <40 (Ref) | 1.00 |  | 1.00 |  | 1.00 |  | 1.00 |  |
| 40-46 | 1.00 (0.862, 1.166) |  | 0.99 (0.857, 1.154) | 0.941 | 0.98 (0.855, 1.118) | 0.744 | 0.98 (0.858, 1.124) | 0.791 |
| >46 | 1.07 (0.915, 1.245) | 0.409 | 1.07 (0.919, 1.246) | 0.383 | 1.04 (0.906, 1.190) | 0.588 | 1.03 (0.900, 1.184) | 0.650 |
| Age | 1.01 (1.003, 1.015) | 0.003 | 1.01 (1.003, 1.015) | 0.003 | 1.00 (0.994, 1.006) | 0.969 | 1.00 (0.990, 1.006) | 0.605 |
| Sex (women) | 0.99 (0.877, 1.127) | 0.923 | 0.98 (0.863, 1.106) | 0.717 | 0.93 (0.833, 1.043) | 0.220 | 0.95 (0.848, 1.069) | 0.403 |
| HbA1c | 1.16 (1.092, 1.238) | <0.001 |  |  | 1.16 (1.096, 1.230) | <0.001 | 1.15 (1.079, 1.215) | <0.001* |
| DM duration | 1.02 (1.014, 1.025) | <0.001 |  |  | 1.02 (1.014, 1.026) | <0.001 | 1.02 (1.013, 1.026) | <0.001* |
| BMI | 1.01 (0.999, 1.029) | 0.072 |  |  | 1.01 (0.999, 1.027) | 0.068 | 1.01 (0.998, 1.026) | 0.096 |
| Physical activity (sedentary) | 0.94 (0.819, 1.084) | 0.405 |  |  | 0.98 (0.866, 1.118) | 0.803 | 0.98 (0.859, 1.110) | 0.719 |
| Smoking | 1.05 (0.929, 1.192) | 0.426 |  |  |  |  | 1.05 (0.932, 1.173) | 0.449 |
| Hypertension | 1.37 (1.189, 1.573) | <0.001 |  |  |  |  | 1.17 (1.003, 1.358) | 0.047* |
| Dyslipidemia | 1.13 (0.999, 1.287) | 0.053 |  |  |  |  | 1.01 (0.886, 1.150) | 0.885 |
| GFR | 1.00 (0.992, 1.001) | 0.183 |  |  |  |  | 1.00 (0.996, 1.006) | 0.604 |

The odds ratios (ORs) with their 95% confidence intervals (CIs) are shown. BMI, body mass index; DM, diabetes mellitus; GFR, glomerular filtration rate; HbA1c: glycosylated hemoglobin; OR, odds ratio; CI, confidence interval. Model 1: adjusted for age and sex. Model 2: adjusted for age, sex, plus HbA1c, DM duration, BMI and sedentary physical activity. Model 3: adjusted for the variables of model 2 plus smoking, hypertension, dyslipidemia and GFR.

**Supplementary Table 10.** Multiple logistic regression models between niacin equivalents intake and mild diabetic retinopathy (No DR vs. Mild DR), before and after adjustment for sex, age, clinical variables, and comorbidities in type 1 diabetes mellitus.

| **Mild DR (T1D)** | | | | | | | | |
| --- | --- | --- | --- | --- | --- | --- | --- | --- |
|  | **Unadjusted model** |  | **Model 1** |  | **Model 2** |  | **Model 3** |  |
| **Niacin equivalents intake (mg/day)** | **OR (CI)** | **p-value** | **OR (CI)** | **p-value** |  | **p-value** |  | **p-value** |
| <40 (Ref) | 1.00 (ref) |  | 1.00 (ref) |  | 1.00 (ref) |  | 1.00 (ref) |  |
| 40-46 | 1.44 (0.694, 2.995) | 0.327 | 1.35 (0.644, 2.839) | 0.426 | 1.24 (0.542, 2.820) | 0.614 | 1.26 (0.547, 2.916) | 0.584 |
| ≥46 | 1.76 (0.836, 3.694) | 0.137 | 1.72 (0.813, 3.639) | 0.156 | 1.57 (0.688, 3.577) | 0.284 | 1.52 (0.662, 3.499) | 0.323 |
| Age | 1.03 (1.002, 1.060) | 0.039 | 1.03 (1.001, 1.060) | 0.044 | 1.00 (0.959, 1.031) | 0.769 | 0.99 (0.946, 1.040) | 0.730 |
| Sex (women) | 1.01 (0.562, 1.812) | 0.976 | 0.94 (0.517, 1.711) | 0.841 | 0.69 (0.354, 1.360) | 0.287 | 0.75 (0.376, 1.486) | 0.406 |
| HbA1c | 1.60 (1.130, 2.256) | <0.001* |  |  | 1.79 (1.225, 2.628) | 0.003* | 1.74 (1.182, 2.567) | 0.005* |
| DM duration | 1.09 (1.051, 1.126) | <0.001* |  |  | 1.10 (1.059, 1.150) | <0.001* | 1.10 (1.058, 1.151) | <0.001* |
| BMI | 1.05 (0.976, 1.132) | 0.190 |  |  | 1.07 (0.981, 1.168) | 0.125 | 1.07 (0.974, 1.164) | 0.165 |
| Physical activity (sedentary) | 0.92 (0.481, 1.749) | 0.793 |  |  | 0.98 (0.461, 2.059) | 0.946 | 0.94 (0.435, 2.009) | 0.864 |
| Smoking | 1.33 (0.738, 2.380) | 0.346 |  |  |  |  | 1.46 (0.740, 2.877) | 0.276 |
| Hypertension | 2.38 (1.166, 4.838) | 0.017 |  |  |  |  | 1.52 (0.613, 3.761) | 0.367 |
| Dyslipidemia | 1.46 (0.806, 2.655) | 0.211 |  |  |  |  | 1.07 (0.490, 2.317) | 0.873 |
| GFR | 1.00 (0.975, 1.018) | 0.717 |  |  |  |  | 1.01 (0.980, 1.042) | 0.505 |

The odds ratios (ORs) with their 95% confidence intervals (CIs) are shown. BMI, body mass index; DM, diabetes mellitus; GFR, glomerular filtration rate; HbA1c: glycosylated hemoglobin; OR, odds ratio; CI, confidence interval. Model 1: adjusted for age and sex. Model 2: adjusted for age, sex, plus HbA1c, DM duration, BMI and sedentary physical activity. Model 3: adjusted for the variables of model 2 plus smoking, hypertension, dyslipidemia and GFR. No DR, no diabetic retinopathy [1]; Mild DR [ETDRS stages 2].

**Supplementary Table 11.** Multiple logistic regression models for association between niacin equivalents intake and advanced diabetic retinopathy (No DR vs. Advanced DR), before and after adjustment for sex, age, clinical variables, and comorbidities in type 1 diabetes mellitus.

| **Advanced DR (T1D)** | | | | | | | | |
| --- | --- | --- | --- | --- | --- | --- | --- | --- |
|  | **Unadjusted model** |  | **Model 1** |  | **Model 2** |  | **Model 3** |  |
| **Niacin equivalents intake (mg/day)** | **OR (CI)** | **p-value** | **OR (CI)** | **p-value** |  | **p-value** |  | **p-value** |
| <40 (Ref) | 1.00 (ref) |  | 1.00 (ref) |  | 1.00 (ref) |  | 1.00 (ref) |  |
| 40-46 | 0.63 (0.250, 1.590) | 0.328 | 0.70 (0.268, 1.811) | 0.458 | 0.75 (0.218, 2.556) | 0.641 | 0.59 (0.158, 2.216) | 0.436 |
| ≥46 | 0.94 (0.385, 2.291) | 0.890 | 0.98 (0.390, 2.455) | 0.963 | 1.07 (0.334, 3.413) | 0.912 | 0.83 (0.243, 2.834) | 0.767 |
| Age | 1.06 (1.017, 1.097) | 0.005 | 1.06 (1.016, 1.096) | 0.006 | 1.02 (0.964, 1.078) | 0.502 | 0.98 (0.910, 1.051) | 0.543 |
| Sex (women) | 0.92 (0.434, 1.951) | 0.829 | 0.89 (0.408, 1.929) | 0.763 | 0.69 (0.257, 1.871) | 0.469 | 0.79 (0.267, 2.316) | 0.663 |
| HbA1c | 2.82 (1.783, 4.459) | <0.001* |  |  | 3.73 (2.147, 6.460) | <0.001* | 3.38 (1.892, 6.046) | <0.001* |
| DM duration | 1.11 (1.064, 1.162) | <0.001* |  |  | 1.13 (1.068, 1.200) | <0.001* | 1.13 (1.060, 1.200) | <0.001* |
| BMI | 1.09 (0.998, 1.180) | 0.057 |  |  | 1.13 (1.005, 1.262) | 0.040 | 1.13 (0.996, 1.275) | 0.059 |
| Physical activity (sedentary) | 0.60 (0.242, 1.490) | 0.272 |  |  | 0.62 (0.180, 2.141) | 0.450 | 0.52 (0.127, 2.120) | 0.361 |
| Smoking | 1.08 (0.508, 2.275) | 0.851 |  |  |  |  | 1.13 (0.380, 3.375) | 0.823 |
| Hypertension | 8.57 (3.734, 19.677) | <0.001* |  |  |  |  | 5.48 (1.697, 17.710) | 0.004* |
| Dyslipidemia | 2.16 (1.010, 4.604) | 0.047 |  |  |  |  | 0.99 (0.306, 3.225) | 0.991 |
| GFR | 0.97 (0.945, 0.996) | 0.024 |  |  |  |  | 1.00 (0.956, 1.040) | 0.882 |

The odds ratios (ORs) with their 95% confidence intervals (CIs) are shown. BMI, body mass index; DM, diabetes mellitus; GFR, glomerular filtration rate; HbA1c: glycosylated hemoglobin; OR, odds ratio; CI, confidence interval. Model 1: adjusted for age and sex. Model 2: adjusted for age, sex, plus HbA1c, DM duration, BMI and sedentary physical activity. Model 3: adjusted for the variables of model 2 plus smoking, hypertension, dyslipidemia and GFR. No DR, no diabetic retinopathy [1]; advanced DR [ETDRS stages 3–5].

**Supplementary Table 12.** Multiple logistic regression models for association between niacin equivalents intake and diabetic retinopathy (Mild DR vs. Advanced DR), before and after adjustment for sex, age, clinical variables, and comorbidities in type 1 diabetes mellitus.

| **Mild DR vs Advanced DR (T1D)** | | | | | | | | |
| --- | --- | --- | --- | --- | --- | --- | --- | --- |
|  | **Unadjusted model** |  | **Model 1** |  | **Model 2** |  | **Model 3** |  |
| **Niacin equivalents intake (mg/day)** | **OR (CI)** | **p-value** | **OR (CI)** | **p-value** |  | **p-value** |  | **p-value** |
| <40 (Ref) | 1.00 (ref) |  | 1.00 (ref) |  | 1.00 (ref) |  | 1.00 (ref) |  |
| 40-46 | 0.44 (0.155, 1.236) | 0.119 | 0.39 (0.135, 1.139) | 0.085 | 0.26 (0.078, 0.847) | 0.026 | 0.21 (0.059, 0.776) | 0.019 |
| ≥46 | 0.53 (0.196, 1.455) | 0.220 | 0.58 (0.209, 1.608) | 0.296 | 0.53 (0.173, 1.634) | 0.270 | 0.46 (0.136, 1.542) | 0.207 |
| Age | 1.02 (0.984, 1.063) | 0.263 | 1.03 (0.986, 1.072) | 0.188 | 1.02 (0.974, 1.076) | 0.352 | 0.99 (0.930, 1.058) | 0.809 |
| Sex (women) | 0.91 (0.399, 2.087) | 0.828 | 0.83 (0.346, 1.966) | 0.664 | 0.49 (0.176, 1.346) | 0.165 | 0.42 (0.125, 1.381) | 0.152 |
| HbA1c | 1.63 (1.096, 2.419) | 0.016 |  |  | 2.29 (1.405, 3.745) | 0.001 | 2.28 (1.361, 3.830) | 0.002* |
| DM duration | 1.02 (0.982, 1.068) | 0.271 |  |  | 1.05 (0.991, 1.105) | 0.104 | 1.04 (0.983, 1.102) | 0.166 |
| BMI | 1.05 (0.950, 1.151) | 0.361 |  |  | 1.09 (0.974, 1.221) | 0.132 | 1.07 (0.941, 1.220) | 0.296 |
| Physical activity (sedentary) | 0.66 (0.244, 1.757) | 0.401 |  |  | 0.57 (0.182, 1.792) | 0.337 | 0.60 (0.176, 2.036) | 0.411 |
| Smoking | 0.81 (0.355, 1.854) | 0.619 |  |  |  |  | 0.47 (0.144, 1.525) | 0.209 |
| Hypertension | 3.61 (1.519, 8.574) | 0.004* |  |  |  |  | 3.86 (1.159, 12.874) | 0.028* |
| Dyslipidemia | 1.47 (0.644, 3.377) | 0.359 |  |  |  |  | 0.70 (0.219, 2.240) | 0.549 |
| GFR | 0.97 (0.938, 1.001) | 0.055 |  |  |  |  | 0.99 (0.941, 1.034) | 0.562 |

The odds ratios (ORs) with their 95% confidence intervals (CIs) are shown. BMI, body mass index; DM, diabetes mellitus; GFR, glomerular filtration rate; HbA1c: glycosylated hemoglobin; OR, odds ratio; CI, confidence interval. Model 1: adjusted for age and sex. Model 2: adjusted for age, sex, plus HbA1c, DM duration, BMI and sedentary physical activity. Model 3: adjusted for the variables of model 2 plus smoking, hypertension, dyslipidemia and GFR. Mild DR [ETDRS stages 2]; advanced DR [ETDRS stages 3–5].

**Supplementary Table 13.** Multiple logistic regression models for association between dietary niacin intake and diabetic retinopathy before and after adjusting for patient sex, age, clinical variables and comorbidities of type 2 diabetes mellitus.

| **Retinopathy** | | | | | | | | |
| --- | --- | --- | --- | --- | --- | --- | --- | --- |
|  | **Unadjusted model** |  | **Model 1** |  | **Model 2** |  | **Model 3** |  |
| Niacin intake (mg/day) | OR (CI) | *p*-value | OR (CI) | *p*-value |  | *p*-value |  | *p*-value |
| <26 (Ref) | 1.00 |  | 1.00 |  | 1.00 |  | 1.00 |  |
| 26-31 | 0.96 (0.829, 1.103) | 0.543 | 0.96 (0.830, 1.103) | 0.540 | 0.97 (0.850, 1.102) | 0.622 | 0.98 (0.854, 1.116) | 0.723 |
| ≥31 | 1.04 (0.905, 1.196) | 0.576 | 1.04 (0.905, 1.196) | 0.574 | 1.01 (0.884, 1.142) | 0.944 | 1.02 (0.897, 1.166) | 0.737 |
| Age | 1.01 (1.001, 1.013) | 0.024 | 1.01 (1.001, 1.013) | 0.026 | 1.00 (0.996, 1.008) | 0.496 | 1.00 (0.993, 1.008) | 0.947 |
| Sex (women) | 1.02 (0.906, 1.141) | 0.773 | 1.01 (0.897, 1.132) | 0.901 | 0.90 (0.805, 1.013) | 0.084 | 0.90 (0.785, 1.030) | 0.126 |
| HbA1c | 1.14 (1.091, 1.180) | <0.001 |  |  | 1.09 (1.049, 1.137) | <0.001 | 1.09 (1.046, 1.136) | <0.001* |
| DM duration | 1.02 (1.017, 1.029) | <0.001 |  |  | 1.02 (1.011, 1.026) | <0.001 | 1.02 (1.011, 1.025) | <0.001* |
| BMI | 1.01 (0.994, 1.016) | 0.357 |  |  | 1.01 (0.995, 1.016 | 0.320 | 1.00 (0.991, 1.013) | 0.696 |
| Physical activity (sedentary) | 1.02 (0.907, 1.142) | 0.765 |  |  | 1.04 (0.939, 1.160) | 0.429 | 1.05 (0.940, 1.162) | 0.419 |
| Smoking | 0.94 (0.838, 1.055) | 0.294 |  |  |  |  | 0.97 (0.851, 1.104) | 0.636 |
| Hypertension | 1.19 (1.061, 1.336) | 0.003 |  |  |  |  | 1.15 (1.018, 1.290) | 0.025* |
| Dyslipidemia | 1.08 (0.964, 1.214) | 0.181 |  |  |  |  | 1.02 (0.908, 1.135) | 0.792 |
| GFR | 1.00 (0.993, 1.001) | 0.130 |  |  |  |  | 1.00 (0.996, 1.005) | 0.828 |

The odds ratios (ORs) with their 95% confidence intervals (CIs) are shown. BMI, body mass index; DM, diabetes mellitus; GFR, glomerular filtration rate; HbA1c, glycosylated hemoglobin; OR, odds ratio; CI, confidence interval. Model 1: adjusted for age and sex. Model 2: adjusted for age, sex, plus HbA1c, DM duration, BMI and sedentary physical activity. Model 3: adjusted for the variables of model 2 plus smoking, hypertension, dyslipidemia and GFR.

**Supplementary Table 14.** Multiple logistic regression models for association between niacin intake and mild diabetic retinopathy (No DR vs. Mild DR), before and after adjustment for sex, age, clinical variables, and comorbidities in type 2 diabetes mellitus.

| **Mild DR (T2D)** | | | | | | | | |
| --- | --- | --- | --- | --- | --- | --- | --- | --- |
|  | **Unadjusted model** |  | **Model 1** |  | **Model 2** |  | **Model 3** |  |
| **Niacin intake (mg/day)** | **OR (CI)** | **p-value** | **OR (CI)** | **p-value** |  | **p-value** |  | **p-value** |
| <26 (Ref) | 1.00 (ref) |  | 1.00 (ref) |  | 1.00 (ref) |  | 1.00 (ref) |  |
| 26-31 | 0.83 (0.378, 1.839) | 0.652 | 0.81 (0.365, 1.793) | 0.602 | 0.74 (0.304, 1.775) | 0.493 | 0.68 (0.276, 1.706) | 0.417 |
| ≥31 | 1.51 (0.730, 3.134) | 0.266 | 1.46 (0.698, 3.034) | 0.316 | 1.17 (0.509, 2.690) | 0.711 | 1.17 (0.498, 2.737) | 0.722 |
| Age | 1.02 (0.985, 1.048) | 0.304 | 1.02 (0.985, 1.049) | 0.301 | 1.00 (0.961, 1.034) | 0.851 | 1.00 (0.955, 1.052) | 0.939 |
| Sex (women) | 0.84 (0.452, 1.545) | 0.567 | 0.84 (0.448, 1.561) | 0.575 | 0.57 (0.270, 1.199) | 0.138 | 0.54 (0.222, 1.324) | 0.179 |
| HbA1c | 1.61 (1.246, 2.088) | <0.001* |  |  | 1.42 (1.079, 1.875) | 0.012 | 1.37 (1.026, 1.838) | 0.033 |
| DM duration | 1.11 (1.058, 1.163) | <0.001* |  |  | 1.11 (1.048, 1.170) | <0.001* | 1.11 (1.047, 1.170) | <0.001* |
| BMI | 1.02 (0.960, 1.083) | 0.525 |  |  | 1.06 (0.982, 1.134) | 0.145 | 1.04 (0.965, 1.122) | 0.304 |
| Physical activity (sedentary) | 1.03 (0.558, 1.892) | 0.931 |  |  | 1.14 (0.569, 2.264) | 0.720 | 1.22 (0.602, 2.473) | 0.580 |
| Smoking | 0.89 (0.482, 1.643) | 0.709 |  |  |  |  | 0.84 (0.359, 1.948) | 0.678 |
| Hypertension | 1.78 (0.954, 3.348) | 0.070 |  |  |  |  | 1.68 (0.777, 3.633) | 0.188 |
| Dyslipidemia | 1.73 (0.931, 3.193) | 0.083 |  |  |  |  | 1.50 (0.728, 3.077) | 0.273 |
| GFR | 1.00 (0.979, 1.023) | 0.932 |  |  |  |  | 1.02 (0.986, 1.056) | 0.247 |

The odds ratios (ORs) with their 95% confidence intervals (CIs) are shown. BMI, Body Mass Index; DM, Diabetes Mellitus; GFR, glomerular filtration rate; HbA1c, glycosylated hemoglobin; OR, odds ratio; CI, confidence interval. Model 1: adjusted for age and sex. Model 2: adjusted for age, sex, plus HbA1c, DM duration, BMI and sedentary physical activity. Model 3: adjusted for the variables of model 2 plus smoking, hypertension, dyslipidemia and GFR. No DR, no diabetic retinopathy [1]; Mild DR [ETDRS stages 2].

**Supplementary Table 15.** Multiple logistic regression models for association between niacin intake and advanced diabetic retinopathy (No DR vs. Advanced DR), before and after adjustment for sex, age, clinical variables, and comorbidities in type 2 diabetes mellitus.

| **Advanced DR (T2D)** | | | | | | | | |
| --- | --- | --- | --- | --- | --- | --- | --- | --- |
|  | **Unadjusted model** |  | **Model 1** |  | **Model 2** |  | **Model 3** |  |
| **Niacin intake (mg/day)** | **OR (CI)** | **p-value** | **OR (CI)** | **p-value** |  | **p-value** |  | **p-value** |
| <26 (Ref) | 1.00 (ref) |  | 1.00 (ref) |  | 1.00 (ref) |  | 1.00 (ref) |  |
| 26-31 | 0.90 (0.463, 1.737) | 0.746 | 0.95 (0.485, 1.876) | 0.892 | 0.95 (0.422, 2.140) | 0.903 | 1.03 (0.432, 2.433) | 0.954 |
| ≥31 | 1.05 (0.544, 2.031) | 0.882 | 1.13 (0.573, 2.228) | 0.724 | 0.91 (0.394, 2.088) | 0.819 | 1.00 (0.418, 2.398) | 0.999 |
| Age | 1.04 (1.008, 1.067) | 0.011 | 1.04 (1.008, 1.067) | 0.013 | 1.02 (0.984, 1.060) | 0.259 | 1.00 (0.951, 1.041) | 0.832 |
| Sex (women) | 1.37 (0.797, 2.361) | 0.255 | 1.35 (0.771, 2.357) | 0.294 | 0.59 (0.285, 1.233) | 0.162 | 0.58 (0.233, 1.463) | 0.251 |
| HbA1c | 2.03 (1.576, 2.614) | <0.001* |  |  | 1.73 (1.312, 2.269) | <0.001* | 1.82 (1.359, 2.436) | <0.001* |
| DM duration | 1.15 (1.099, 1.202) | <0.001* |  |  | 1.12 (1.066, 1.178) | <0.001* | 1.11 (1.059, 1.172) | <0.001* |
| BMI | 1.03 (0.980, 1.087) | 0.237 |  |  | 1.05 (0.972, 1.126) | 0.233 | 1.03 (0.948, 1.109) | 0.535 |
| Physical activity (sedentary) | 1.14 (0.660, 1.953) | 0.646 |  |  | 1.28 (0.651, 2.531) | 0.470 | 1.29 (0.643, 2.587) | 0.473 |
| Smoking | 0.69 (0.399, 1.183) | 0.176 |  |  |  |  | 0.89 (0.379, 2.104) | 0.795 |
| Hypertension | 2.10 (1.196, 3.698) | 0.010 |  |  |  |  | 1.91 (0.883, 4.110) | 0.101 |
| Dyslipidemia | 1.28 (0.741, 2.194) | 0.380 |  |  |  |  | 0.88 (0.430, 1.806) | 0.730 |
| GFR | 0.98 (0.959, 0.995) | 0.012 |  |  |  |  | 0.98 (0.954, 1.010) | 0.204 |

The odds ratios (ORs) with their 95% confidence intervals (CIs) are shown. BMI, Body Mass Index; DM, Diabetes Mellitus; GFR, glomerular filtration rate; HbA1c, glycosylated hemoglobin; OR, odds ratio; CI, confidence interval. Model 1: adjusted for age and sex. Model 2: adjusted for age, sex, plus HbA1c, DM duration, BMI and sedentary physical activity. Model 3: adjusted for the variables of model 2 plus smoking, hypertension, dyslipidemia and GFR. No DR, no diabetic retinopathy [1]; advanced DR [ETDRS stages 3–5].

**Supplementary Table 16.** Multiple logistic regression models for association between niacin intake and diabetic retinopathy (Mild DR vs. Advanced DR), before and after adjustment for sex, age, clinical variables, and comorbidities in type 2 diabetes mellitus.

| **Mild DR vs Advanced DR (T2D)** | | | | | | | | |
| --- | --- | --- | --- | --- | --- | --- | --- | --- |
|  | **Unadjusted model** |  | **Model 1** |  | **Model 2** |  | **Model 3** |  |
| **Niacin intake (mg/day)** | **OR (CI)** | **p-value** | **OR (CI)** | **p-value** |  | **p-value** |  | **p-value** |
| <26 (Ref) | 1.00 (ref) |  | 1.00 (ref) |  | 1.00 (ref) |  | 1.00 (ref) |  |
| 26-31 | 1.08 (0.453, 2.557) | 0.869 | 1.11 (0.463, 2.664) | 0.815 | 1.16 (0.469, 2.855) | 0.751 | 1.09 (0.416, 2.837) | 0.866 |
| ≥31 | 0.70 (0.313, 1.544) | 0.372 | 0.76 (0.335, 1.701) | 0.498 | 0.72 (0.308, 1.684) | 0.449 | 0.69 (0.277, 1.703) | 0.417 |
| Age | 1.03 (0.986, 1.065) | 0.205 | 1.02 (0.979, 1.061) | 0.348 | 1.02 (0.977, 1.069) | 0.341 | 0.99 (0.931, 1.046) | 0.645 |
| Sex (women) | 1.64 (0.834, 3.227) | 0.151 | 1.46 (0.724, 2.953) | 0.290 | 1.25 (0.543, 2.880) | 0.600 | 1.49 (0.505, 4.400) | 0.469 |
| HbA1c | 1.21 (0.946, 1.555) | 0.128 |  |  | 1.18 (0.906, 1.545) | 0.216 | 1.25 (0.942, 1.647) | 0.124 |
| DM duration | 1.03 (0.993, 1.067) | 0.109 |  |  | 1.01 (0.965, 1.051) | 0.751 | 1.00 (0.955, 1.043) | 0.929 |
| BMI | 1.01 (0.952, 1.076) | 0.704 |  |  | 1.01 (0.937, 1.080) | 0.865 | 1.01 (0.936, 1.090) | 0.788 |
| Physical activity (sedentary) | 1.11 (0.564, 2.164) | 0.770 |  |  | 1.09 (0.533, 2.215) | 0.819 | 0.96 (0.454, 2.021) | 0.910 |
| Smoking | 0.77 (0.394, 1.512) | 0.451 |  |  |  |  | 1.18 (0.441, 3.131) | 0.746 |
| Hypertension | 1.18 (0.581, 2.383) | 0.650 |  |  |  |  | 0.96 (0.400, 2.312) | 0.930 |
| Dyslipidemia | 0.74 (0.377, 1.452) | 0.381 |  |  |  |  | 0.64 (0.296, 1.396) | 0.264 |
| GFR | 0.97 (0.951, 0.998) | 0.035 |  |  |  |  | 0.96 (0.930, 1.000) | 0.053 |

The odds ratios (ORs) with their 95% confidence intervals (CIs) are shown. BMI, Body Mass Index; DM, Diabetes Mellitus; GFR, glomerular filtration rate; HbA1c, glycosylated hemoglobin; OR, odds ratio; CI, confidence interval. Model 1: adjusted for age and sex. Model 2: adjusted for age, sex, plus HbA1c, DM duration, BMI and sedentary physical activity. Model 3: adjusted for the variables of model 2 plus smoking, hypertension, dyslipidemia and GFR. Mild DR [ETDRS stages 2]; advanced DR [ETDRS stages 3–5].


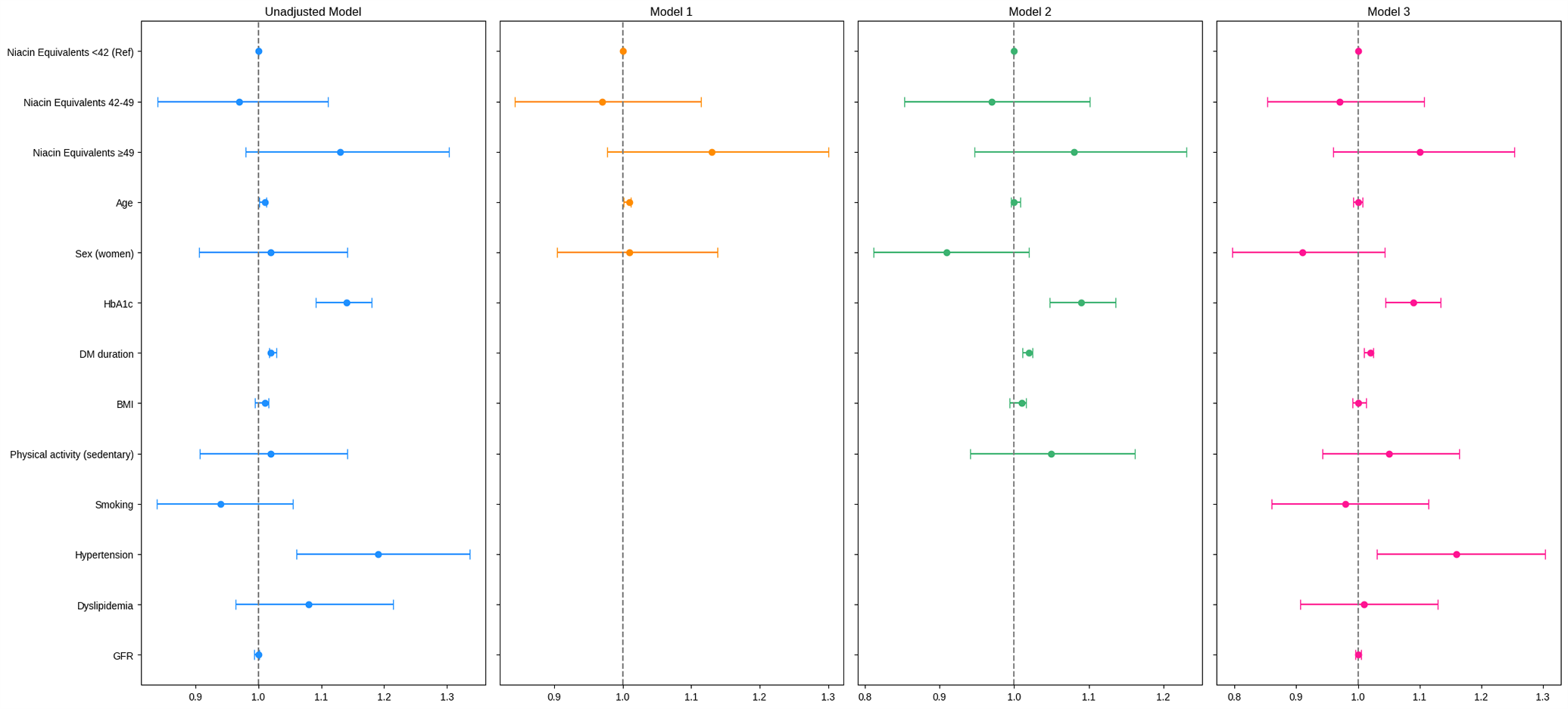


**Supplementary Figure 7.** Forest plot of multiple logistic regression of dietary niacin equivalent intake (mg/day) in type 2 diabetes mellitus group with diabetic retinopathy.

The odds ratios (ORs) with their 95% confidence intervals (CIs) are represented. BMI, body mass index; DM, diabetes mellitus; GFR, glomerular filtration rate; HbA1c, glycosylated hemoglobin. Model 1: adjusted for age and sex. Model 2: adjusted for age, sex, plus HbA1c, DM duration, BMI and sedentary physical activity. Model 3: adjusted for the variables of model 2 plus smoking, hypertension, dyslipidemia and GFR. The association between DR and niacin intake was calculated with the relative effect measure of odds ratios (ORs) and the 95% confidence interval (CI).

**Supplementary Table 17.** Multiple logistic regression models for association between niacin equivalents intake and diabetic in type 2 diabetes mellitus group before and after adjusting for patient sex, age, clinical variables and comorbidities.

| **Retinopathy** | | | | | | | | |
| --- | --- | --- | --- | --- | --- | --- | --- | --- |
|  | **Unadjusted model** |  | **Model 1** |  | **Model 2** |  | **Model 3** |  |
| Niacin equivalents intake (mg/day) | OR (CI) | *p*-value | OR (CI) | *p*-value |  | *p*-value |  | *p*-value |
| <42 (Ref) | 1.00 |  | 1.00 |  | 1.00 |  | 1.00 |  |
| 42-49 | 0.97 (0.840, 1.111) | 0.627 | 0.97 (0.843, 1.114) | 0.659 | 0.97 (0.853, 1.101) | 0.632 | 0.97 (0.853, 1.107) | 0.670 |
| >49 | 1.13 (0.980, 1.303) | 0.095 | 1.13 (0.977, 1.300) | 0.102 | 1.08 (0.947, 1.231) | 0.255 | 1.10 (0.960, 1.254) | 0.173 |
| Age | 1.01 (1.001, 1.013) | 0.024 | 1.01 (1.001, 1.012) | 0.034 | 1.00 (0.996, 1.008) | 0.535 | 1.00 (0.993, 1.008) | 0.948 |
| Sex (women) | 1.02 (0.906, 1.141) | 0.773 | 1.01 (0.904, 1.138) | 0.813 | 0.91 (0.812, 1.020) | 0.108 | 0.91 (0.796, 1.044) | 0.181 |
| HbA1c | 1.14 (1.091, 1.180) | <0.001 |  |  | 1.09 (1.048, 1.136) | <0.001 | 1.09 (1.045, 1.134) | <0.001* |
| DM duration | 1.02 (1.017, 1.029) | <0.001 |  |  | 1.02 (1.011, 1.025) | <0.001 | 1.02 (1.010, 1.025) | <0.001* |
| BMI | 1.01 (0.994, 1.016) | 0.357 |  |  | 1.01 (0.994, 1.016) | 0.346 | 1.00 (0.991, 1.013) | 0.781 |
| Physical activity (sedentary) | 1.02 (0.907, 1.142) | 0.765 |  |  | 1.05 (0.941, 1.162) | 0.406 | 1.05 (0.943, 1.164) | 0.390 |
| Smoking | 0.94 (0.838, 1.055) | 0.294 |  |  |  |  | 0.98 (0.860, 1.114) | 0.747 |
| Hypertension | 1.19 (1.061, 1.336) | 0.003 |  |  |  |  | 1.16 (1.031, 1.304) | 0.014* |
| Dyslipidemia | 1.08 (0.964, 1.214) | 0.181 |  |  |  |  | 1.01 (0.906, 1.130) | 0.834 |
| GFR | 1.00 (0.993, 1.001) | 0.130 |  |  |  |  | 1.00 (0.996, 1.005) | 0.730 |

The odds ratios (ORs) with their 95% confidence intervals (CIs) are shown. BMI, body mass index; DM, diabetes mellitus; GFR, glomerular filtration rate; HbA1c, glycosylated hemoglobin; OR, odds ratio; CI, confidence interval. Model 1: adjusted for age and sex. Model 2: adjusted for age, sex, plus HbA1c, DM duration, BMI and sedentary physical activity. Model 3: adjusted for the variables of model 2 plus smoking, hypertension, dyslipidemia and GFR.

**Supplementary Table 18.** Multiple logistic regression models for association between niacin equivalents intake and mild diabetic retinopathy (No DR vs. Mild DR), before and after adjustment for sex, age, clinical variables, and comorbidities in type 2 diabetes mellitus.

| **Mild DR (T2D)** | | | | | | | | |
| --- | --- | --- | --- | --- | --- | --- | --- | --- |
|  | **Unadjusted model** |  | **Model 1** |  | **Model 2** |  | **Model 3** |  |
| **Niacin equivalents intake (mg/day)** | **OR (CI)** | **p-value** | **OR (CI)** | **p-value** |  | **p-value** |  | **p-value** |
| <42 (Ref) | 1.00 (ref) |  | 1.00 (ref) |  | 1.00 (ref) |  | 1.00 (ref) |  |
| 42-49 | 0.73 (0.331, 1.613) | 0.437 | 0.72 (0.324, 1.591) | 0.415 | 0.63 (0.262, 1.519) | 0.304 | 0.59 (0.236, 1.454) | 0.249 |
| ≥49 | 1.98 (0.940, 4.187) | 0.072 | 1.90 (0.893, 4.031) | 0.096 | 1.52 (0.649, 3.575) | 0.334 | 1.61 (0.666, 3.907) | 0.289 |
| Age | 1.02 (0.985, 1.048) | 0.304 | 1.01 (0.982, 1.045) | 0.419 | 0.99 (0.958, 1.031) | 0.748 | 1.00 (0.953, 1.051) | 0.971 |
| Sex (women) | 0.84 (0.452, 1.545) | 0.567 | 0.84 (0.447, 1.574) | 0.584 | 0.57 (0.269, 1.210) | 0.144 | 0.58 (0.235, 1.435) | 0.239 |
| HbA1c | 1.61 (1.246, 2.088) | <0.001* |  |  | 1.42 (1.083, 1.869) | 0.011 | 1.37 (1.023, 1.829) | 0.034 |
| DM duration | 1.11 (1.058, 1.163) | <0.001* |  |  | 1.11 (1.046, 1.170) | <0.001* | 1.11 (1.044, 1.169) | 0.001* |
| BMI | 1.02 (0.960, 1.083) | 0.525 |  |  | 1.05 (0.980, 1.132) | 0.161 | 1.04 (0.960, 1.118) | 0.366 |
| Physical activity (sedentary) | 1.03 (0.558, 1.892) | 0.931 |  |  | 1.09 (0.543, 2.181) | 0.811 | 1.18 (0.576, 2.394) | 0.658 |
| Smoking | 0.89 (0.482, 1.643) | 0.709 |  |  |  |  | 0.94 (0.397, 2.213) | 0.883 |
| Hypertension | 1.79 (0.954, 3.348) | 0.070 |  |  |  |  | 1.78 (0.808, 3.915) | 0.152 |
| Dyslipidemia | 1.73 (0.931, 3.193) | 0.083 |  |  |  |  | 1.53 (0.737, 3.172) | 0.255 |
| GFR | 1.00 (0.979, 1.023) | 0.933 |  |  |  |  | 1.02 (0.988, 1.059) | 0.200 |

The odds ratios (ORs) with their 95% confidence intervals (CIs) are shown. BMI, Body Mass Index; DM, Diabetes Mellitus; GFR, glomerular filtration rate; HbA1c, glycosylated hemoglobin; OR, odds ratio; CI, confidence interval. Model 1: adjusted for age and sex. Model 2: adjusted for age, sex, plus HbA1c, DM duration, BMI and sedentary physical activity. Model 3: adjusted for the variables of model 2 plus smoking, hypertension, dyslipidemia and GFR. No DR, no diabetic retinopathy [1]; Mild DR [ETDRS stages 2].

**Supplementary Table 19.** Multiple logistic regression models for association between niacin equivalents intake and advanced diabetic retinopathy (No DR vs. Advanced DR), before and after adjustment for sex, age, clinical variables, and comorbidities in type 2 diabetes mellitus.

| **Advanced DR (T2D)** | | | | | | | | |
| --- | --- | --- | --- | --- | --- | --- | --- | --- |
|  | **Unadjusted model** |  | **Model 1** |  | **Model 2** |  | **Model 3** |  |
| **Niacin equivalents intake (mg/day)** | **OR (CI)** | **p-value** | **OR (CI)** | **p-value** |  | **p-value** |  | **p-value** |
| <42 (Ref) | 1.00 (ref) |  | 1.00 (ref) |  | 1.00 (ref) |  | 1.00 (ref) |  |
| 42-49 | 0.89 (0.462, 1.719) | 0.731 | 0.90 (0.463, 1.764) | 0.768 | 0.81 (0.358, 1.811) | 0.601 | 0.87 (0.375, 2.030) | 0.752 |
| ≥49 | 1.45 (0.732, 2.860) | 0.288 | 1.52 (0.756, 3.047) | 0.241 | 1.38 (0.588, 3.238) | 0.459 | 1.58 (0.645, 3.845) | 0.319 |
| Age | 1.04 (1.008, 1.067) | 0.011 | 1.04 (1.008, 1.067) | 0.013 | 1.02 (0.984, 1.060) | 0.259 | 1.00 (0.951, 1.042) | 0.834 |
| Sex (women) | 1.37 (0.797, 2.361) | 0.255 | 1.39 (0.796, 2.429) | 0.246 | 0.60 (0.288, 1.261) | 0.179 | 0.60 (0.237, 1.493) | 0.269 |
| HbA1c | 2.03 (1.576, 2.614) | <0.001* |  |  | 1.70 (1.289, 2.237) | <0.001* | 1.79 (1.336, 2.406) | <0.001* |
| DM duration | 1.15 (1.099, 1.202) | <0.001* |  |  | 1.12 (1.067, 1.182) | <0.001* | 1.12 (1.061, 1.178) | <0.001* |
| BMI | 1.03 (0.980, 1.087) | 0.237 |  |  | 1.05 (0.976, 1.130) | 0.186 | 1.03 (0.950, 1.110) | 0.508 |
| Physical activity (sedentary) | 1.14 (0.660, 1.953) | 0.646 |  |  | 1.37 (0.691, 2.698) | 0.371 | 1.38 (0.685, 2.768) | 0.369 |
| Smoking | 0.69 (0.399, 1.183) | 0.176 |  |  |  |  | 0.90 (0.382, 2.113) | 0.807 |
| Hypertension | 2.10 (1.196, 3.698) | 0.010 |  |  |  |  | 2.11 (0.961, 4.611) | 0.063 |
| Dyslipidemia | 1.28 (0.741, 2.194) | 0.380 |  |  |  |  | 0.88 (0.430, 1.785) | 0.716 |
| GFR | 0.98 (0.959, 0.995) | 0.012 |  |  |  |  | 0.98 (0.956, 1.013) | 0.282 |

The odds ratios (ORs) with their 95% confidence intervals (CIs) are shown. BMI, Body Mass Index; DM, Diabetes Mellitus; GFR, glomerular filtration rate; HbA1c, glycosylated hemoglobin; OR, odds ratio; CI, confidence interval. Model 1: adjusted for age and sex. Model 2: adjusted for age, sex, plus HbA1c, DM duration, BMI and sedentary physical activity. Model 3: adjusted for the variables of model 2 plus smoking, hypertension, dyslipidemia and GFR. No DR, no diabetic retinopathy [1]; advanced DR [ETDRS stages 3–5].

**Supplementary Table 20.** Multiple logistic regression models for association between niacin equivalents intake and diabetic retinopathy (Mild DR vs. Advanced DR), before and after adjustment for sex, age, clinical variables, and comorbidities in type 2 diabetes mellitus.

| **Mild DR vs Advanced DR (T2D)** | | | | | | | | |
| --- | --- | --- | --- | --- | --- | --- | --- | --- |
|  | **Unadjusted model** |  | **Model 1** |  | **Model 2** |  | **Model 3** |  |
| **Niacin equivalents intake (mg/day)** | **OR (CI)** | **p-value** | **OR (CI)** | **p-value** |  | **p-value** |  | **p-value** |
| <42 (Ref) | 1.00 (ref) |  | 1.00 (ref) |  | 1.00 (ref) |  | 1.00 (ref) |  |
| 42-49 | 1.22 (0.508, 2.930) | 0.656 | 1.26 (0.519, 3.069) | 0.608 | 1.26 (0.502, 3.163) | 0.623 | 1.24 (0.472, 3.248) | 0.663 |
| ≥49 | 0.73 (0.325, 1.637) | 0.444 | 0.77 (0.339, 1.742) | 0.528 | 0.77 (0.328, 1.799) | 0.544 | 0.74 (0.303, 1.808) | 0.510 |
| Age | 1.03 (0.986, 1.065) | 0.205 | 1.02 (0.981, 1.063) | 0.306 | 1.02 (0.979, 1.071) | 0.306 | 0.99 (0.931, 1.046) | 0.645 |
| Sex (women) | 1.64 (0.834, 3.227) | 0.151 | 1.45 (0.719, 2.931) | 0.299 | 1.26 (0.551, 2.900) | 0.580 | 1.40 (0.467, 4.200) | 0.547 |
| HbA1c | 1.21 (0.946, 1.555) | 0.128 |  |  | 1.18 (0.901, 1.544) | 0.231 | 1.24 (0.939, 1.646) | 0.129 |
| DM duration | 1.03 (0.993, 1.067) | 0.109 |  |  | 1.01 (0.965, 1.050) | 0.759 | 1.00 (0.955, 1.043) | 0.932 |
| BMI | 1.01 (0.952, 1.076) | 0.704 |  |  | 1.01 (0.937, 1.080) | 0.870 | 1.01 (0.937, 1.091) | 0.777 |
| Physical activity (sedentary) | 1.11 (0.564, 2.164) | 0.770 |  |  | 1.10 (0.542, 2.236) | 0.789 | 0.97 (0.464, 2.038) | 0.941 |
| Smoking | 0.77 (0.394, 1.512) | 0.451 |  |  |  |  | 1.06 (0.396, 2.818) | 0.913 |
| Hypertension | 1.18 (0.581, 2.383) | 0.650 |  |  |  |  | 0.99 (0.416, 2.331) | 0.972 |
| Dyslipidemia | 0.74 (0.377, 1.452) | 0.381 |  |  |  |  | 0.64 (0.299, 1.389) | 0.262 |
| GFR | 0.97 (0.951, 0.998) | 0.035 |  |  |  |  | 0.96 (0.929, 0.999) | 0.044 |

The odds ratios (ORs) with their 95% confidence intervals (CIs) are shown. BMI, Body Mass Index; DM, Diabetes Mellitus; GFR, glomerular filtration rate; HbA1c, glycosylated hemoglobin; OR, odds ratio; CI, confidence interval. Model 1: adjusted for age and sex. Model 2: adjusted for age, sex, plus HbA1c, DM duration, BMI and sedentary physical activity. Model 3: adjusted for the variables of model 2 plus smoking, hypertension, dyslipidemia and GFR. Mild DR [ETDRS stages 2]; advanced DR [ETDRS stages 3–5].
